# Citric acid and itaconic acid accumulation: variations of the same story?

**DOI:** 10.1007/s00253-018-09607-9

**Published:** 2019-02-13

**Authors:** Levente Karaffa, Christian P. Kubicek

**Affiliations:** 1 Department of Biochemical Engineering, Faculty of Science and Technology, University of Debrecen, Egyetem tér 1, Debrecen, H-4032 Hungary; 2 Institute of Chemical, Environmental & Bioscience Engineering, TU Wien, Getreidemarkt 9, 1060 Vienna, Austria; 3 Present Address: 1100 Vienna, Austria

**Keywords:** *Aspergillus niger*, *Aspergillus terreus*, Citric acid, Itaconic acid, Submerged fermentation, Overflow metabolism

## Abstract

Citric acid production by *Aspergillus niger* and itaconic acid production by *Aspergillus terreus* are two major examples of technical scale fungal fermentations based on metabolic overflow of primary metabolism. Both organic acids are formed by the same metabolic pathway, but whereas citric acid is the end product in *A*. *niger*, *A*. *terreus* performs two additional enzymatic steps leading to itaconic acid. Despite of this high similarity, the optimization of the production process and the mechanism and regulation of overflow of these two acids has mostly been investigated independently, thereby ignoring respective knowledge from the other. In this review, we will highlight where the similarities and the real differences of these two processes occur, which involves various aspects of medium composition, metabolic regulation and compartmentation, transcriptional regulation, and gene evolution. These comparative data may facilitate further investigations of citric acid and itaconic acid accumulation and may contribute to improvements in their industrial production.

**Electronic supplementary material:**

The online version of this article (10.1007/s00253-018-09607-9) contains supplementary material, which is available to authorized users.

## Introduction

Citric acid (2-hydroxy-propane-1,2,3-tricarboxylic acid) and itaconic acid (2-methylene-succinic acid or 2-methylidenebutanedioic acid) are the most well-known examples of fungal “overflow metabolism,” a term coined by Foster (1949) to describe the seemingly wasteful strategy of some fungi to incompletely oxidize their carbon source. The first fungal production process for citric acid employing *Aspergillus niger* is a century old (Currie 1917). Itaconic acid accumulation by *Aspergillus itaconicus* was described in the early 30s of the last century (Kinoshita 1931), while the first production technology—already employing *Aspergillus terreus*—was patented in the next decade (Kane et al. 1945). Before World War II, organic acid manufacturing was exclusively performed by the labor-intensive and relatively low-yield surface method (Doelger and Prescott 1934; Calam et al. 1939). In this process, fungi were grown in aluminum trays of 50–200 cm depth, filled up with sugar and inorganic nutrients, with sterile air—serving as an oxygen supply and a cooling agent—being passed over them. Submerged organic acid fermentation employing agitated, aerated, high-quality steel vessels started on the sidelines of penicillin manufacturing (Kane et al. 1945; Perlman et al. 1946a, b; Lockwood and Nelson 1946; Martin and Waters 1952). Commercial large-scale submerged fermentations by *A*. *niger* (for citric acid) and *A*. *terreus* (for itaconic acid) have been initiated all across the world in the 50s (Steel et al. 1955). Although some citric acid is produced by solid-state fermentation, the estimated 2.1 million tons of citric acid manufactured annually is almost completely performed by the submerged method, most typically batch wise (Cavallo et al. 2017). Itaconic acid is exclusively produced this way (Bafana and Pandey 2018). Despite the intense research on lab- and pilot scale (Yu et al. 2018), no continuous or immobilized cell-based fermentation methods are used on technical scale for any of the two organic acids. In summary, from the technological point of view (scale, vessel types, inoculation protocols, critical process parameters, medium composition), the upstream parts as well as kinetics of product and biomass formation of these two industrial fermentations are very much similar (Supplementary Tables S1 and S2).

Citric acid is mainly used as a flavoring agent in the food industry, and to a lesser extent as an acidifier and a chelating agent in the chemical and pharma industries (Apelblat 2014). Itaconic acid is produced in significantly less amounts (41.000 t/a in 2011; Geiser et al. 2016), but the actual market potential is estimated at 80.000 t/a. Indeed, in 2004, the US Department of Energy assigned itaconic acid as one of the top 12 most promising building block chemicals for bio-based economy (Werpy and Petersen 2004). Due to the conjugated double bond of the methylene group, its fundamental application is being a precursor for the polymer industry (absorbents, unsaturated polyester resins, plastics; Okabe et al. 2009; Kuenz and Krull 2018). Importantly, itaconic acid could in principle replace polyacrylic acid whose production is petroleum-based. Should that happen completely, a market worth over $11 billion would open up (El-Imam and Du 2014; Saha et al. 2018), making itaconic acid one of the crown jewels of industrial biotechnology. However, that would require at least 25% fall in prices that currently stands at around $2/kg, preventing wider use of this compound.

The biochemistry and production of citric and itaconic acid have recently been subject of several excellent reviews (Steiger et al. 2013; Chen and Nielsen 2016; Bafana and Pandey 2018; Zhao et al. 2018; Kuenz and Krull 2018), but a critical comparison of the similar and dissimilar aspects of their biosynthesis and accumulation has not been made. In this review, we will document that the biochemistry and physiology of accumulation of these two organic acids is indeed almost identical. The only difference is the presence of three additional genes in *A*. *terreus*, whose evolution and origin will be discussed.

## Biological importance of citric acid and itaconic acid

The biological function of the formation of the two acids have never been studied in details, but it is generally assumed that both serve as strong acidifiers and therefore provide selective advance for these two acid-tolerant fungi in their habitat over bacteria and yeast. Another potential function of citric acid (but not itaconic acid) may be its ability to chelate manganese and ferrous ions, thereby participating in their transport into the cell and conversely, in the scavenging of these metal ions from uptake by competing organisms (Guerinot et al. 1990; Odoni et al. 2017). Out-competing rivals for metal ions has been shown to be an important defense strategy in fungi (Canessa and Larrondo 2013; Blatzer and Latgé 2017).

In contrast to citric acid, itaconic acid itself has been described as a weapon to combat competitors: itaconic acid has been identified in activated pro-inflammatory macrophages, and its effect on cellular metabolism has been attributed to the inhibition of succinate dehydrogenase (Strelko et al. 2011; Michelucci et al. 2013). Furthermore, itaconate was shown to induce electrophilic stress through reacting with glutathione and subsequently inducing the transcription factor Nrf2 which is essential for protection against oxidative and xenobiotic stresses, and attenuate inflammation (Kobayashi et al. 2013; Bambouskova et al. 2018). Itaconate also inhibits isocitrate lyase (McFadden et al. 1971), a key enzyme of the glyoxylate cycle, required for the survival of bacterial and fungal pathogens inside the eukaryotic host (Sasikaran et al. 2014). Although the role of itaconic acid in the survival of *A*. *terreus* in its natural environment has not been studied yet, we consider reasonable that its antimicrobial effects are beneficial also there, and that this could have an impact on the regulation of its formation.

## Biochemical pathways of citric and itaconic acid biosynthesis

Citric acid is an intermediate of the citric acid cycle, which is an essential part of energy metabolism in all aerobic eukaryotic heterotrophs (Krebs 1940). Cleland and Johnson (1954), using ^14^C-labeled d-glucose pioneered by demonstrating that citric acid accumulation occurs by a condensation of oxaloacetate and pyruvate, the former arising by fixation of the carbon dioxide from one of the two pyruvate molecules derived from glucose during oxidative decarboxylation to acetyl-CoA. By this way, no carbon dioxide is lost what enabled molar yields of citric acid > 66%, the maximum that would arise by the standard operation of the TCA cycle. They also demonstrated that no further metabolism of citric acid by the citric acid cycle occurred, and that no CO_2_ was released by the pentose phosphate pathway.

Shortly thereafter, Bentley and Thiessen (1957) demonstrated that the same pathway operates in *A*. *terreus* with the only exception that citric acid is further metabolized via *cis*-aconitate to itaconate. The operation of this pathway in an industrial strain of *A*. *terreus* was later confirmed by tracer experiments with ^14^C- and ^13^C-labeled substrates (Bonnarme et al. 1995). They also showed that no CO_2_ was released during the production phase, indicating that no glucose metabolism via the pentose phosphate pathway occurred during itaconic acid accumulation either.

**Fig. 1:**
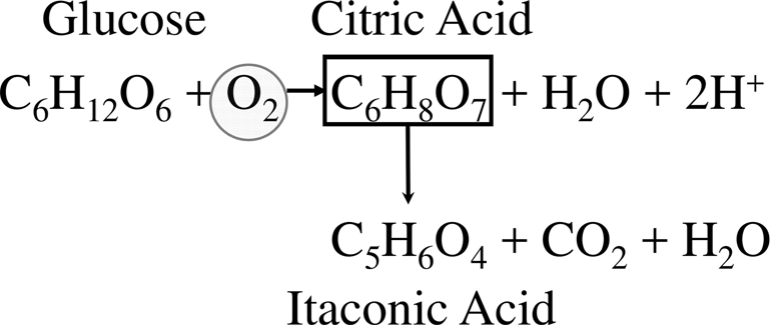
Mass balance of glucose-citrate-itaconate bioconversions

A further point that must be noted is that citrate is a product of incomplete oxidative metabolism, and therefore contains one more oxygen atom than the hexose from which it is formed (Fig. 1). This also applies to itaconic acid, although the overall stoichiometry discloses this because of the removal of carbon dioxide toward the end of its biosynthesis. This requirement of extra oxygen has important implications for the conditions leading to the production of these two acids in high amounts (see below). To sum up, the only metabolic difference between citric acid and itaconic acid biosynthesis is that *A*. *terreus* does not accumulate citrate but metabolizes it to *cis*-aconitate, which is then converted to itaconate by *cis*-aconitate decarboxylase (Steiger et al. 2013).

## Nutrient preconditions for citric acid and itaconic acid production

**Fig. 2:**
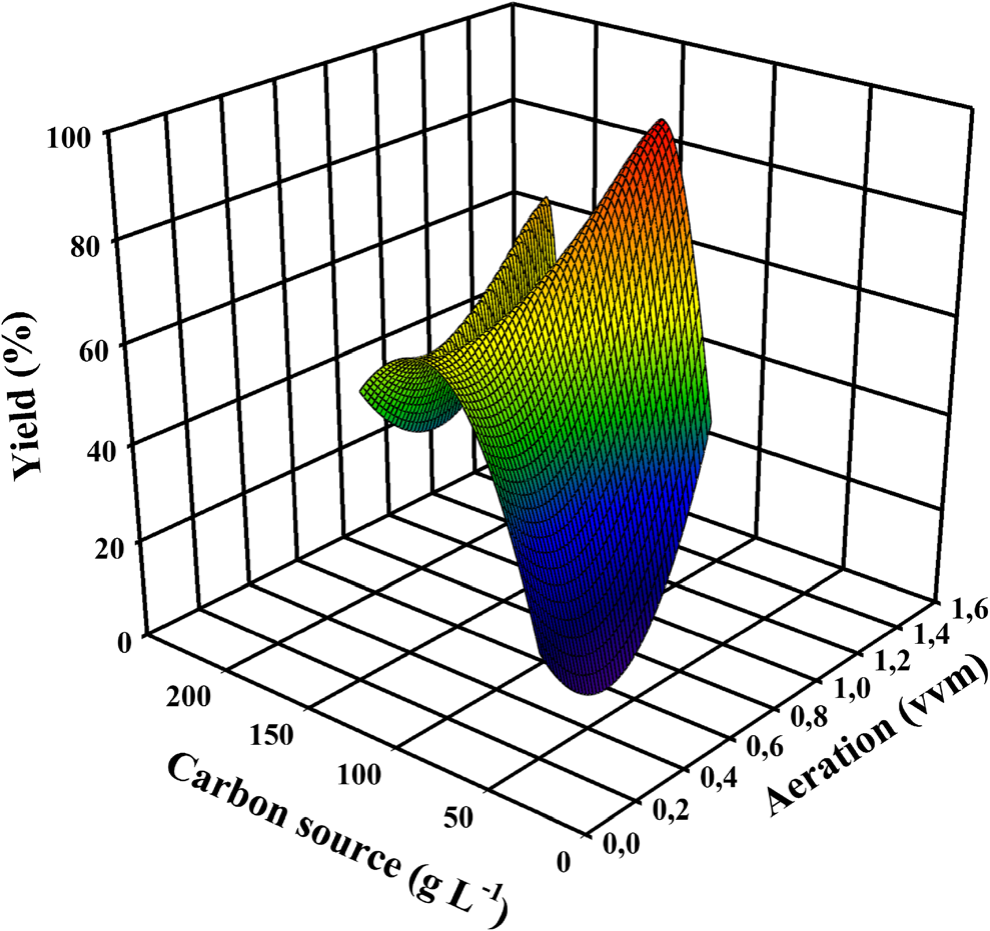
A 3D plot demonstrating the trilateral relationship between citric acid molar yield, aeration of the fermenter, and the concentration of the carbon source used. Input data are taken from well-documented, publicly available fermentations, listed in Supplementary Table S5. Plot was made with SigmaPlot 12.0 (Systat Software, Inc., USA)

**Fig. 3:**
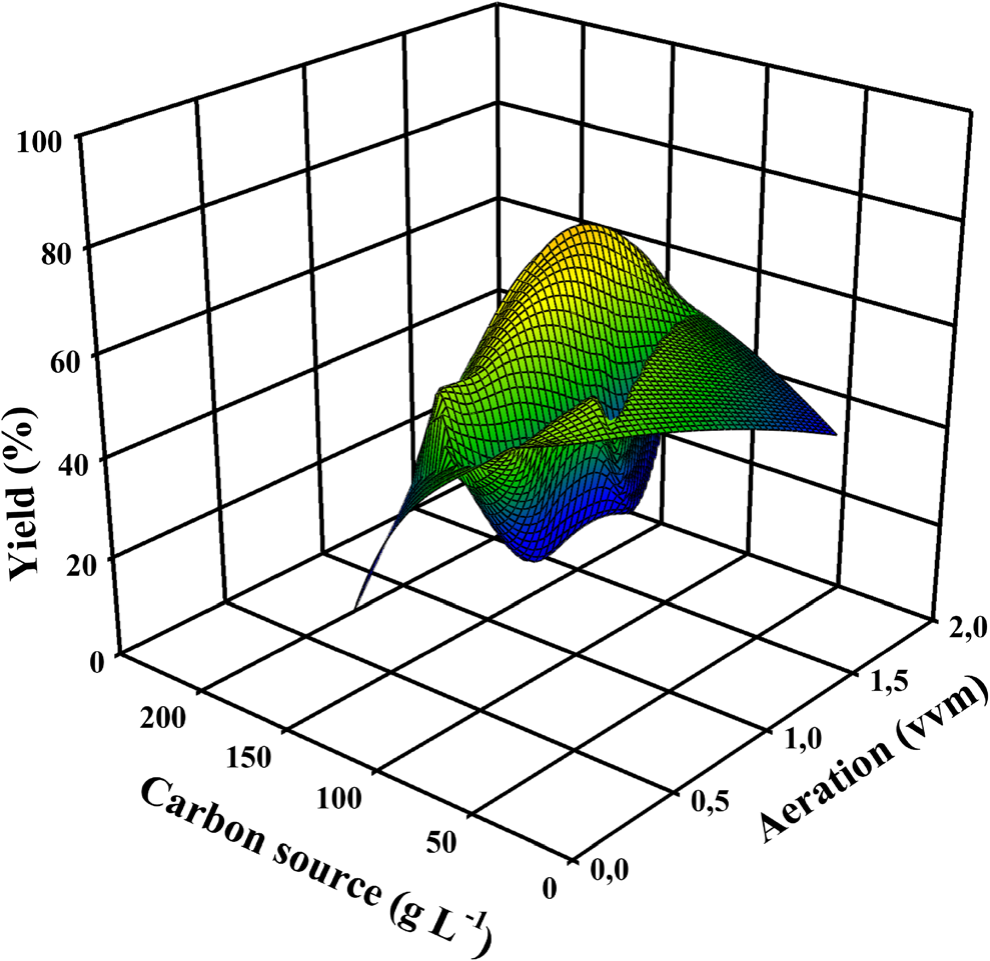
A 3D plot demonstrating the trilateral relationship between itaconic acid molar yield, aeration of the fermenter, and the concentration of the carbon source used. Input data are taken from well-documented, publicly available fermentations, listed in Supplementary Table S6. Plot was made with the same software as above

To discuss similarities and dissimilarities in the production of citric acid and itaconic acid, the first aspect that needs to be compared is the nutrient requirements for these two processes. The fundamental prerequisite for overflow metabolism is a metabolic deregulation that is caused by a combination of abundance of the carbon source and serious shortage of phosphate and some micronutrient(s). Yet there are other, critical factors that determine whether the accumulation of citric and itaconic acid reaches levels beyond a molar *Y*_p/s_ > 0.7 (i.e., by a process involving pyruvate carboxylation, see above). Studies that fulfilled this criterion showed that the carbon source must be a rapidly metabolizable hexose or disaccharide and be supplied in concentrations > 100 g/L. This high concentration of the hexose obviously also requires a correspondingly high degree of aeration to fulfill the stoichiometry of citric acid/itaconic acid biosynthesis (see above; Molnár et al. 2018; Figs. 2 and 3). The concentration of inorganic nutrients should be sufficient to enable biomass formation in amounts of 10–15 g dry weight/L, which enables the production of both acids in the range of yields defined above.

In addition, both acids have been proven to be accumulated in the respective high concentrations only if the pH of the medium is below 3. There are some data in the literature that itaconic acid can also be produced at a higher pH (Nubel and Ratajak 1962; Hevekerl et al. 2014), but these studies did not reach the high itaconic acid concentrations we consider essential for this review. In *A*. *niger*, the requirement for a low pH has been explained by the need to bypass gluconic acid formation by the extracellular glucose oxidase which is inactivated by a pH < 3 (Kubicek and Karaffa 2010), but *A*. *terreus* is not known to produce gluconic acid. The reason for the requirement of a low pH must therefore be due to different, so far unknown reasons.

**Fig. 4:**
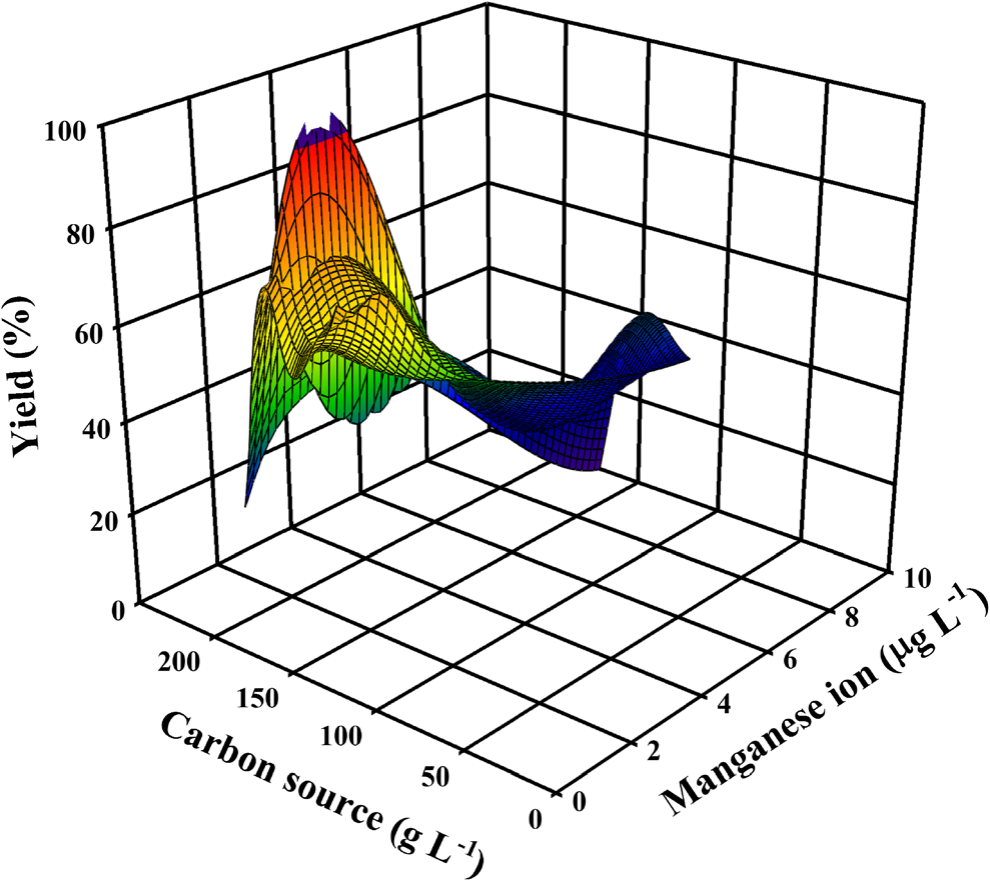
A 3D plot demonstrating the trilateral relationship between citric acid molar yield, Mn(II) ion concentration, as well as carbon source concentration used. Input data are taken from well-documented, publicly available fermentations, listed in Supplementary Table S7. Plot was made with the same software as above

Conflicting information is available about the role of metal ions for citric and itaconic acid formation, respectively. In the case of *A*. *niger*, US and Canadian research groups led by Johnson and Clark, respectively, have demonstrated that high yields can only be obtained when the concentration of Mn(II) ions in the medium is below 4 μg/L (Clark 1962; Fig. 4). A similar inhibitory effect has been demonstrated for Fe(II) at concentrations > 2 mg/L (Tomlinson et al. 1950). The concentration of Zn(II) did not affect citric acid formation. Interestingly, the inhibition of citric acid accumulation by iron has attracted more attention in literature. In our opinion, this has two main reasons: one is that purification of molasses by the addition of hexacyanoferrate—a classical method to remove ferrous ions—has been proven in industry to be necessary to achieve high citric acid yields. The other one is that the first enzyme that performs downstream metabolism of citrate in the cell—aconitase—is an iron-containing enzyme, and it was believed for a long time that iron limitation in the medium leads to a blockage of aconitase activity and thus triggers citric acid overflow (Ramakrishnan et al. 1955). Yet this hypothesis has been clearly falsified by proving that citrate and isocitrate are present in citric acid-producing mycelia of *A*. *niger* in concentrations perfectly reflecting the aconitase equilibrium and the enzyme must therefore be active (Kubicek and Röhr 1985).

How can the effect of iron then be interpreted? The critical point is that metal ions of analytical grade, as used in research, always contain impurities. Iron salts contain up to 0.5% (*w*/*w*) manganese, which implies that the addition of 2 mg/L iron introduces a concentration of 10 μg/L of Mn(II) ions, which—as explained above—would per se be sufficient for a significant inhibition of citric acid formation. In addition, the treatment of molasses with ferrocyanide precipitates not only iron but also manganese and zinc ions. This suggests that the effects reported for iron are in fact due to the presence of manganese ions, which therefore is the critical parameter to obtain high yield of citrate.

**Fig. 5:**
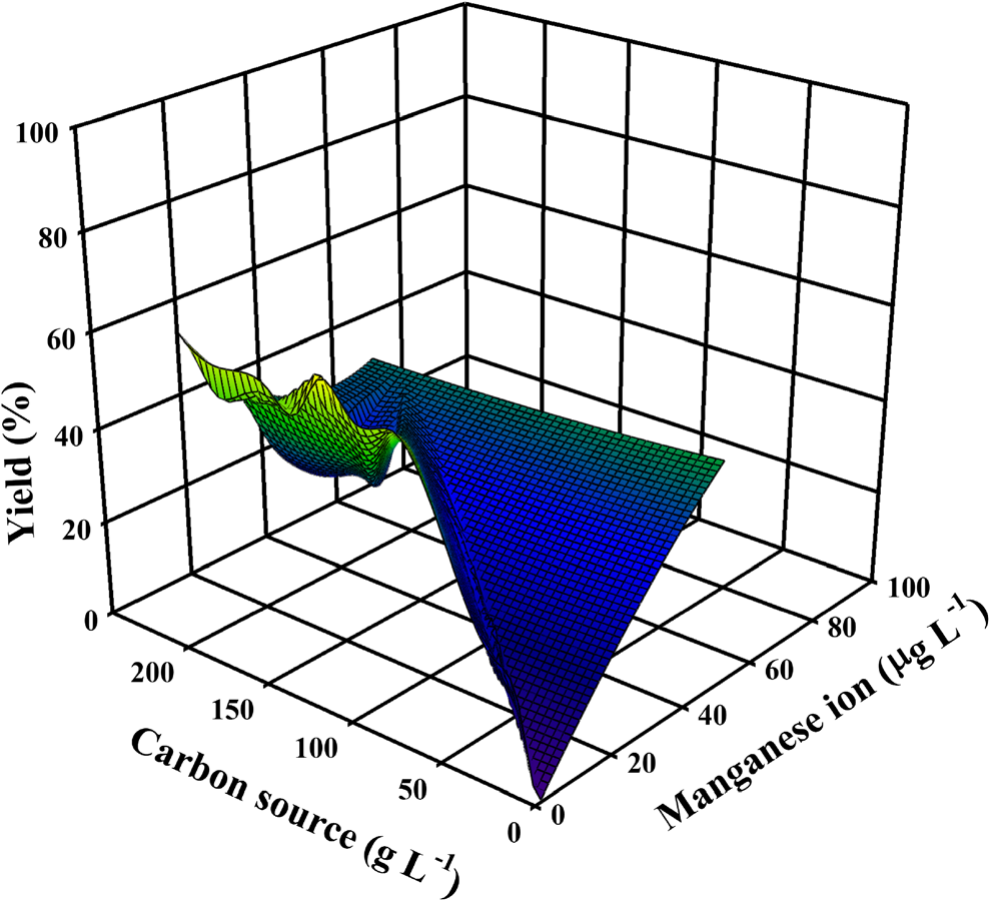
A 3D plot demonstrating the trilateral relationship between itaconic acid molar yield, Mn(II) ion concentration, as well as carbon source concentration used. Input data are taken from well-documented, publicly available fermentations, listed in Supplementary Table S8. Plot was made with the same software as above

With regard to itaconic acid accumulation, comparably few studies in this direction have been published. Relevant literature regarding the effect of Mn(II) ions is summarized in Fig. 5. Ferrocyanide treatment was shown to enhance itaconic acid production (Batti and Schweiger 1963). Karaffa et al. (2015) provided evidence that the overproduction of itaconic acid requires a medium in which the Mn(II) concentration is in the same range as in citric acid fermentations. Interestingly, however, there are also reports on itaconic acid fermentations with high yields which do not attempt to remove metal ions (Shin et al. 2013, 2017). This issue therefore needs further investigation.

## Morphology

The above described manganese deficiency also leads to a typical restricted morphology which consists of small pellets (< 0.5 mm diameter) with branches of short hyphae with swollen tips (Snell and Schweiger 1949; Takahashi et al. 1958; Takahashi and Yamada 1959). Image analysis showed that citric acid molar yields exceed 85% when the macro-morphological form is constituted by stable particles of intertwined filaments around a small nucleus, while the micro-morphology is characterized by branches of short hyphae with swollen tips (Cox et al. 1998; Papagianni and Mattey 2006). A similar morphology was developed by *A*. *terreus* during high-yield itaconate production (Batti and Schweiger 1963; Gyamerah 1995; Kuenz et al. 2012). Karaffa et al. (2015) showed that Mn(II) deficiency is the critical factor determining this morphology also in *A*. *terreus*. Thus, the influence of manganese and other heavy metal ions on the morphology is similar in citric acid and itaconic acid fermentations. The background of this requirement is believed to be the decreased viscosity of the fermentation broth, which results in improved oxygen transfer and increased solute uptake.

There have been few attempts only to genetically stabilize the development of the small pellets, and they have so far been done with *A*. *niger* only: the main cell wall of *A*. *niger* contains β-1,3-glucan, chitin, β-1,6-glucan, α-1,3-glucan, galactosaminogalactan, and galactomannan (Pel et al. 2007). The cell walls of the pellets that are formed under manganese-deficient conditions have an increased chitin and lowered ß-glucan content (Kisser et al. 1980). Consistent with this, Yin et al. (2017) found that expression of chitin synthase C (*chsC*) and *pstA*, encoding a cell surface glycosylphosphatidylinositol-anchored protein important for cell wall integrity and resistance against low pH (Pardo et al. 2004; Gil-Bona et al. 2015), were highly upregulated during citric acid fermentation. Sun et al. (2018) silenced *chsC* in an industrial mutant strain which resulted in strains with altered morphology and with lower proportion of dispersed mycelia, which caused the desired decrease in viscosity, improved oxygen, and mass transfer efficiency and improved citric acid production. Dai et al. (2004) applied a different strategy (suppression subtractive hybridization) to identify genes that are differentially expressed under manganese sufficient and deficient conditions, and identified a gene in *A*. *niger* (brsa25 = An03g00640), which encodes an unknown amino acid permease whose antisense inhibition permitted pelleted growth and increased citrate production also in the presence of high manganese concentrations.

## Evolution of the itaconate biosynthesis cluster

**Fig. 6:**
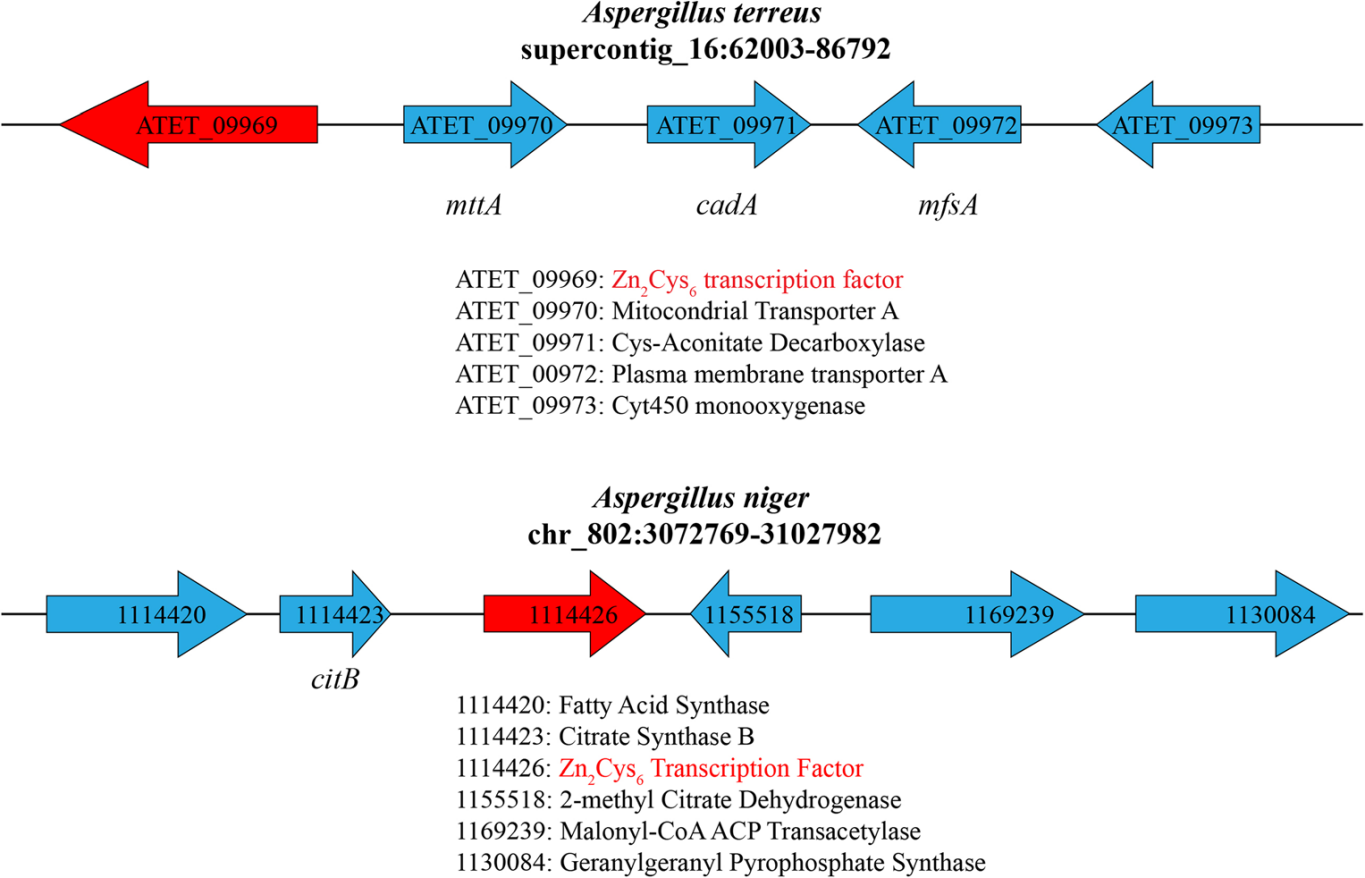
Genomic environment of the Zn_2_Cys_6_ transcriptional activator present in the itaconate biosynthesis cluster of *A*. *terreus* and in *A*. *niger*. Genes are specified by the protein ID used in *A*. *terreus* NIH 2624 (https://genome.jgi.doe.gov/Aspte1/Aspte1.home.html) and *A*. *niger* ATCC 1015 (https://genome.jgi.doe.gov/Aspni7/Aspni7.home.html). Italic abbreviations specify the genes whose function has been experimentally verified

The identification of the genes encoding the necessary steps leading from citrate to itaconate in the *A*. *terreus* genome was a breakthrough in the understanding of itaconic acid biosynthesis. Using differential expression, Li et al. (2011a, b) identified four genes—one (abbreviation still lacking) encoding a Zn_2_Cys_6_-type transcriptional regulator, *mttA* (encoding a mitochondrial *cis*-aconitate transporter), *cadA* (*cis*-aconitate decarboxylase), and *mfsA* encoding a membrane transporter of the major facilitator subfamily—that occur adjacent to the cluster for the biosynthesis of the polyketide lovastatin (Fig. 6). Two further genes—*cypA* (encoding a cytochrome p450 monooxygenase) and *rdoA* (encoding an α-ketoglutarate-(FeII)-dependent dioxygenase)—are located at the 3′ side of the itaconic acid cluster. They could be involved in the conversion of itaconic acid to 2-hydroxiparaconic acid which involves further oxidation steps (Guevarra and Tabuchi 1990). It is tempting to speculate that itaconic acid formation originated from this cluster, e.g., by decreasing the expression of *cypA* and *rdoA.* Unfortunately, the expression of these two genes has not been studied under itaconic acid-producing conditions yet. In this review, we therefore use the term “itaconate gene cluster” only for the four genes first identified by Li et al. (2011a, b). *CadA* and *mttA* were among the most strongly expressed under the conditions used by the authors. Interestingly, functional verification of these four genes by gene deletion is still lacking, and experimental evidence for the role of *cadA*, *mttA*, and *mfsA* in itaconic acid biosynthesis has been provided only by introducing them into *A*. *niger* which lacks *cadA* and *mttA* and therefore does not produce itaconic acid. Consequently, the introduction of *cadA* alone lead only to very low titers of itaconic acid formation, whereas the introduction of *cadA* and *mttA* led to the much higher titers (Li et al. 2011a, b; van der Straat et al. 2014; van der Straat and de Graaff 2014), indicating that these are the critical elements discriminating the citric acid and itaconic acid overflow. Introduction of *mfsA* (either with *cadA* or together with *cadA* and *mttA*) did not lead to increased production levels of itaconic acid, implying that an endogenous *A*. *niger* transporter can efficiently transport itaconate. Interestingly, expression of *mfsA* in *A*. *niger* led to increased citrate production, suggesting that MfsA may be a transporter with broad substrate specificity. Broad substrate specificity was demonstrated for many transporters of the MFS superfamily (Yan 2013) and MfsA orthologs are present in several other Aspergilli who do not produce itaconic acid (Supplementary Table S3). It would therefore be interesting to investigate the phenotype of an *mfsA* knock-out strain of *A*. *terreus* with respect to itaconic acid production.

**Fig. 7:**
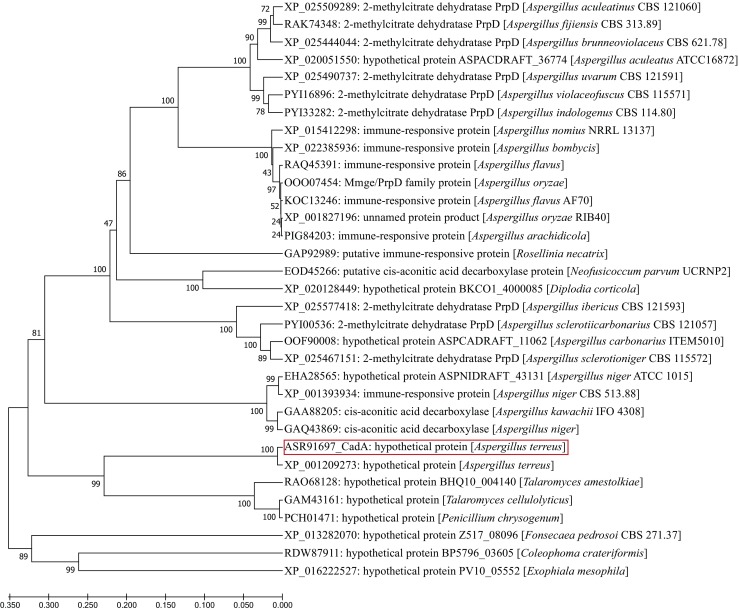
Maximum likelihood tree of *A*. *terreus* CadA (*cis*-aconitate decarboxylase). Proteins used for the tree were identified by a BLASTp search at NCBI, using CadA as bait and the 32 closest hits used for a phylogenetic analysis using maximum likelihood with 1000 bootstrap replicase in MEGA5.0 (https://www.megasoftware.net/). With the exception of CadA, none of the detected proteins has been functionally characterized. The names used in the tree are those submitted by the depositors and should be considered hypothetical

The two essential enzymes for itaconate production—CadA and MttA—seem to be absent from other fungi, because the best hits in NCBI BLASTp are proteins with only low score, low overall similarity, and a high *E* value (Supplementary Table S3). It is therefore interesting that the relatively best hits for MttA were not found in other Aspergilli, but are scattered throughout different other fungal classes and families. This could either indicate that MttA arose by horizontal gene transfer or that an ancient progenor of the *mttA* gene was lost in all other Aspergilli. In contrast, the best hits for CadA—although of low score, identity, and high *E* values—were exclusively from Aspergilli and Penicillia. Interestingly, most of them were annotated as methylcitrate dehydratases, which are members of the same protein family (MmgE/PrpD) as CadA. The close similarity of these dehydratases with carboxylase is puzzling: the decarboxylation reaction catalyzed by CadA is unique among carboxy-lyases because it catalyzes a decarboxylation resulting in both the reduction of a β-γ unsaturation as well as the formation of a methylidene group α by decarboxylation without removal of any other group (Strelko et al. 2011). A phylogeny of the best hits of CadA in NCBI BLASTp shows that it forms a basal clade to all other putative methylcitrate dehydratases with three undefined Penicillium proteins as a sister clade (Fig. 7). CadA therefore seems to have evolved before the splitting of Aspergillus and Penicillium (200–150 mya; van Steenwyk et al. 2018) and subsequently has been lost in most other species of these two fungal families. The catalytic nature of the progenor of the MmgE/PrpD family remains obscure, however. A *cis-*aconitate decarboxylase has also been identified in mammalian cells (where it is called immune-responsive gene 1, *Irg1*), but the IRG1 protein also catabolizes fatty acid-derived metabolites (Michelucci et al. 2013). Despite catalyzing *cis*-aconitate decarboxylation, no homology could be detected between IRG1 and CadA (L. Karaffa and C.P. Kubicek, unpublished data).

Interestingly, the gene encoding the Zn_2_Cys_6_ transcriptional activator of the *A*. *terreus* “itaconate cluster” is present in other Aspergilli including *A*. *niger* (*E* value 2E-119; 42% identity). In the *A*. *niger* genome, it clusters with genes encoding a cytosolic citrate synthase (*citB*), a methylcitrate dehydratase, a fatty acid synthase, and a putative terpenoid synthase (Fig. 6). Hossain et al. (2016) proposed that this gene cluster may encode the genes for the biosynthesis of tensyuic acid, an alkyl-itaconic acid (Hasegawa et al. 2007). This would imply that *A*. *niger* can form itaconate but obviously by a different mechanism: the gene encoding methylcitrate synthase in this cluster (XP_001393190.1) shares no major similarity with CadA and its close hits in BLASTp all annotate as methylcitrate dehydratase for which activity on *cis*-aconitate has so far not been reported yet. The role of the Zn_2_Cys_6_ transcription factor present in the “itaconic acid” and “tensyuic acid cluster,” with respect to itaconic and citric acid accumulation, clearly needs further studies. Transformation of *Aspergillus nidulans*, who does not produce citric acid, with *A*. *niger citB* has recently been shown to lead to citric acid accumulation (Vesth et al. 2018) thus stressing the potential importance of this gene in this process.

Finally, we should like to note that the genes necessary for the formation of itaconic acid by *Ustilago mayidis* are also organized as a cluster in the genome (Geiser et al. 2016). However, its biosynthesis involves *trans*- not *cis*-aconitate, and BLASTp of the *A*. *terreus* genome with the *U*. *mayidis* cluster genes identifies only genes not occurring in the “itaconate cluster” as best hits (although with less than 41% identity and *E* values > E-040). Since the clusters in these two fungi do not comprise homologs, they are likely the result of convergent evolution.

## Glycolytic flux and the role of phosphofructokinase

The tracer studies emphasized above show that the almost exclusive operation of glycolysis plays a major role in both processes, and that its speed could be rate limiting. Based on studies with *Saccharomyces cerevisiae*, metabolic regulation by allosteric feedback inhibition and feed-forward activation was accepted as a major mechanism controlling the flux through glycolysis in fungi, and 6-phosphofructokinase (PfkA) was considered as the candidate for the strongest control (Fiechter and Gmünder 1989; Entian and Barnett 1992). However, regulation at this step during citric acid production was challenged by the findings that citrate is a metabolic inhibitor of PfkA from yeast (Salas et al. 1965).

The kinetic properties of PfkA have therefore been extensively studied in *A*. *niger*. It is upregulated by fructose-2,6-biphosphate (Fru-2,6-P_2_), AMP, and NH_4_^+^ ions, while ATP and citrate have inhibitory effects on the enzyme (Habison et al. 1983; Arts et al. 1987). Since the intracellular Fru-2,6-P_2_ concentration increased considerably with increasing concentrations of glucose in the medium (Kubicek et al. 1990), Fru-2,6-P_2_ may antagonize citrate inhibition in vivo. This hypothesis is consistent with the findings that the overexpression of *pfkA* gene in *A*. *niger* did not increase citric acid production, but reduced the intracellular concentration of Fru-2,6-P_2_ almost twofold compared to the wild-type strain. Thus, the fungus seems to adapt to overexpression of phosphofructokinase by decreasing the specific activity of the enzyme through a reduction in the level of fructose 2,6-P_2_.

In addition, it was observed (Mlakar and Legiša 2006; Capuder et al. 2009) that PfkA undergoes proteolytic truncation when *A*. *niger* is grown under citric acid-producing conditions, and that this fragment (after phosphorylation by protein kinase A) is not inhibited by citrate. Interestingly, this PfkA fragment is stimulated by Fru-2,6-P_2_ to a higher degree than the native PfkA, thus enabling a higher activity in vivo. Consistent with these interpretations, transformation of *A*. *niger* with this PFK fragment resulted in higher rates of citric acid production (Capuder et al. 2009).

The successful stimulation of citric acid production in *A*. *niger* by a PfkA fragment prompted to test whether this fragment would also increase the rate of itaconic acid production by *A*. *terreus*. Its expression in *A*. *terreus* (Tevz et al. 2010) resulted in an increased rate of itaconate accumulation and a 1.5-fold increase in its final concentration. Unfortunately, however, the parent strain formed only 28 g/L itaconate (*Y*_p/s_ = 0.4). Huang et al. (2014), performing similar experiments, also observed a twofold stimulation of itaconic acid production but only under conditions of low final molar yield (*Y*_p/s_ = 0.15 in the parent strain), whereas no effect was observed in a strain displaying high molar yields (*Y*_p/s_ = 0.9). This difference could be explained by the fact that the latter study used an industrial strain of *A*. *terreus* in which glycolytic flux may already have been enhanced during strain development.

One point not considered so far is why a citrate-insensitive PfkA would lead to increased glycolytic rates during itaconate production at all. During citric acid production by *A*. *niger*, citrate is transported into the cytoplasm where it reaches high levels (Habison et al. 1983). In itaconic acid-producing *A*. *terreus*, however, the canonical view of its biosynthesis assumes that *cis*-aconitate, formed in the mitochondria, is transported into the cytoplasm and consequently citrate should not accumulate to high amounts there. It is of course possible that *cis*-aconitate may also inhibit PfkA, but this has not been investigated yet. Itaconate, however, has been reported to be a potent inhibitor of PfkB (forming Fru-2,6-P_2_; Sakai et al. 2004), and thus the concentration of this major activator of PfkA may become reduced in the presence of itaconate. Since the truncated PfkA has a much higher affinity for Fru-2,6-P_2_ (Mesojednik and Legiša 2005), this would overcome the shortage of this activator and stimulate the rate of itaconic acid formation.

## Are citric acid and itaconic acid completely synthesized in the cytoplasm?

As already described above, the canonical view of biosynthesis of citric and itaconic acid is that the two products of glycolysis—pyruvate and oxaloacetate (the latter formed in the cytosol from pyruvate by carboxylation and transported as malate) enter the mitochondria, and after conversion of pyruvate to acetyl-CoA—are used by citrate synthase to form citric acid. Citric acid is then transported into the cytosol by exchange with malate via the mitochondrial citrate/malate antiporter CtpA. In *A*. *terreus*, citrate is first converted to *cis-*aconitate, which is then exported into the cytosol by the mitochondrial carrier MttA (Hossain et al. 2016). Kirimura et al. (2016) however showed that a *ctpA* knock-out strain of *A*. *niger* still accumulates citric acid with a rate of about 50% of the parental strain. This could be explained by postulating that there may be a second or even more mitochondrial transporters for citric acid. However, the major mitochondrial citrate synthase gene *citA* is next neighbor to the *ctpA* in the *A*. *niger* genome, and citrate synthase of *S*. *cerevisiae* is known to bind CtpA located in the inner mitochondrial membrane (Grigorenko et al. 1990), which suggests that CtpA is the main antiporter transporting citrate into the cytoplasm.

An alternative explanation could be that part of citrate is not synthesized in the mitochondria but in the cytosol. This hypothesis is supported by the presence of two cytosolic isoenzymes of citrate synthase in the cytosol (one being CitB, see above), and by the fact that the overexpression of the mitochondrial citrate synthase CitA in *A*. *niger* did not lead to increased citrate accumulation. Since pyruvate decarboxylase is also located in the cytosol, citrate biosynthesis would then only need a cytosolic supply of acetyl-CoA. One possibility, albeit not studied so far, is the oxidation of pyruvate to acetaldehyde and subsequent conversion to acetyl-CoA by the acetyl-CoA synthase FacA. Both genes are present and expressed in *A*. *niger*, but have not yet been investigated in this regards. Alternatively, cytosolic acetyl-CoA could be formed by ATP-citrate lyase, whose expression is upregulated in *A*. *niger* under high citric acid producing conditions (Chen et al. 2014). This would constitute a futile cycle that converts 1 mol ATP back to ADP and inorganic phosphate. The main benefit of such a mechanism would be the production of less ATP which would be further augmented by alterations in the respiratory chain. The latter aspect and its consequences are described below.

*A*. *terreus* has been reported to have no CitB ortholog (Hossain et al. 2016). BLASTp search of its genome, however, retrieved two citrate synthases with moderate similarity to CitB: ATET_2020 (*E* value 8.3E-43) and ATET_8593 (*E* value 3.7E-30). Analysis by TargetP shows that ATET_2020 bears a mitochondrial import signal, whereas ATET_8593 does not. Thus the latter is likely located in the cytosol and a cytosolic citrate biosynthesis seems therefore to be possible in *A*. *terreus*, too. In fact, when the *A*. *terreus* itaconic acid cluster genes were transformed into *A*. *niger*, the resulting itaconate formation was stimulated by overexpression of *A*. *niger citB* (Hossain et al. 2016). On the other hand, overexpression of the main mitochondrial isoenzyme—CitA—did not result in increased itaconate production (Huang et al. 2014), thus paralleling the results obtained with CitA and citric acid production in *A*. *niger* (Ruijter et al. 2000). In addition, *A*. *terreus* also has three aconitases of which one is predicted to be localized in the cytosol by TargetP (unpublished data), and *cis*-aconitate decarboxylase is only found in the cytosol. Furthermore, an acetaldehyde and an acetyl-CoA synthase are—as in *A*. *niger*—also present, and a complete cytoplasmic pathway would therefore be possible. It is of interest in this regards that Blumhoff et al. (2013) found that expression and transport of the *A*. *terreus* aconitase and *cis*-aconitate decarboxylase into the same cellular compartment in *A*. *niger* leads to higher itaconate production than when they were present in their native compartments. Unfortunately, such investigations have not yet been made with *A*. *terreus* so far and the effect of deleting the gene encoding mitochondrial *cis*-aconitate transporter MttA has not yet been studied either. Its overexpression, however, does not stimulate itaconic acid production, and—in contrast—leads to lower production in some transformants (Huang et al. 2014).

## NADH reoxidation and production of ATP

As noted above, the availability of dissolved oxygen at equimolar concentrations to that of glucose is mandatory for citric acid and itaconic acid accumulation in high yields, which is in clear excess of the (minimal) concentration needed for maximal biomass formation (Karaffa and Kubicek 2003; Kubicek and Karaffa 2010). Since co-enzymes such as NAD^+^ or ATP are present in cells only in limited total amounts, this implies a careful balance of their use and regeneration. The biosynthesis of citrate or itaconate via the canonical pathways produces 2 mol of NADH during glycolysis and a further one during acetyl-CoA formation by the pyruvate dehydrogenase reaction. A strong operation of glycolysis, as occurs during citric acid and itaconic acid fermentation, therefore produces 2 ATP and generates 3 NADH that are converted to additional 6.5 ATP by the operation of the respiratory chain (Table 1). Once growth has stopped, only cell maintenance and pyruvate carboxylase consume ATP, which therefore accumulates and will impair metabolism. It should be noted that the maintenance of the pH gradient between the external medium (pH 1.5) and the cytosol (pH between 6.5 and 7.2; Hesse et al. 2000) also consumes ATP, but the stoichiometry is difficult to calculate because the uptake of nutrients (e.g., glucose) also requires H^+^ for proton symport.

**Table 1:** ATP production during citric acid and itaconic acid biosynthesis under different metabolic scenarios, explained in the text. ATP and NADH calculations have been done based on standard biochemical pathway information. The cytochrome C oxidase step of the respiratory chain has been calculated to lead to 0.5 ATP

			ATP production from NADH by		Canonical citrate/itaconate biosynthesis	The same with 100% alternative respiration	Canonical citrate/itaconate biosynthesis with futile citrate cycling	The same with 100% alternative respiration	Cytoplasmic citrate/itaconate biosynthesis	The same with 100% alternative respiration
			Standard	Alternative						
	ATP	NADH	respiratory chain							
Glycolysis	2	2	5	0	7	2	7	2	2	2
Oxidative decarboxylation		1	2.5	1	2.5	1	2.5	1	0	0
Pyruvate carboxylase	− 1				− 1	− 1	− 1	− 1	− 1	− 1
ATP citrate lyase	− 1						− 1	− 1		
Acetaldehyde dehydrogenase		1	1.5	0					1.5	0
Acetyl-CoA-synthase	− 1								− 1	− 1
			Total ATP/per citric acid synthesis		8.5	2	7.5	1	1.5	0

As a consequence, accumulation of citric and itaconic acid requires alternative mechanisms that are not coupled to ATP generation. One of them is known as the alternative oxidase, which directly oxidizes ubiquinon without proton pumping and consequently without ATP production (Li et al. 2011a, b). It bypasses the cyanide- or antimycin-sensitive steps of the respiratory chain (hence its old name “cyanide-resistant respiration”). It is known to be expressed under conditions of oxidative stress (Abrashev et al. 2008) and thus also during citric and itaconic acid fermentations (Kubicek et al. 1979; Zehentgruber et al. 1980; Kirimura et al. 2016; Molnár et al. 2018). Wallrath et al. (1991) showed that a citric acid-producing mutant strain exhibited reduced activity of the proton-pumping respiratory complexes and the activity of NADH-ubiquinone oxidoreductase was selectively lost at the onset of citric acid production. This loss of NADH-ubiquinon oxidoreductase activity appears to be important for citric acid accumulation because its knock-out resulted in a 20-fold increased concentration of intracellular citric acid, and increased amounts of citrate in the medium (Prömper et al. 1993). Unfortunately, citric acid production reported in this paper was comparably low (40 g/L), and it remains unclear whether these findings would also be valid under overproducing conditions. It nevertheless shows that alternative mechanism for reoxidation of NADH produced in mitochondria must operate during citric acid accumulation.

Wang et al. (2015) extended the above findings by hypothesizing that a reduction in the activity of the later stages of the standard respiratory chain would also stimulate citric acid production under high producing (150–170 g/L) conditions. To this end, they used an inhibitor that blocks ubiquinone-cytochrome C oxidase (antimycin) and the standard uncoupler 2,4-dinitrophenol that dissipates the H^+^ gradient needed for ATP generation. Both treatments resulted in an about 20% increase in citric acid yield, thus confirming that an interruption of the standard respiratory chain is beneficial also under high citric-producing conditions. These findings would imply that the operation of the alternative oxidase is essential for citric acid accumulation. However, Hou et al. (2018) found that strains in which one of the alternative oxidase genes (*aoxA*) had been knocked out displayed only a 25% reduced citric acid yield in a high producing strain of *A*. *niger*. It must be pointed out, however, that there is also a second alternative oxidase gene in *A*. *niger* (*aoxB*), which has not been studied do far.

Another intriguing aspect of the alternative respiratory chain is that it is rapidly deactivated by short interruptions in the air supply leading to irreversible decreases in citric acid formation (Zehentgruber et al. 1980). This phenomenon was observed in itaconic acid fermentations as well (Gyamerah 1995; Lin et al. 2004; Kuenz et al. 2012). Lin et al. (2004) therefore inserted a hemoglobin gene from the Gram-negative bacterium *Vitreoscilla sp.* into the *A*. *terreus* genome which resulted in a small antagonism against an interruption of aeration under moderate (40 g/L) itaconic acid production.

## Regulation of citric acid and itaconic acid accumulation by wide domain regulators

Most of the studies on regulation of formation of these both acids have been done at the level of the metabolic reactions forming and transporting them. No data are so far available whether (and how) they are controlled at a genome wide level. The role of the Zn_2_Cys_6_ transcriptional activator encoded in the “itaconate cluster” (see above) would be an attractive candidate. In fact, the “itaconate cluster” genes were identified because of their strong expression in *A*. *terreus* (Li et al. 2011a, b), and Kanamasa et al. (2008) reported that *cadA* is more strongly expressed in a high producer strain of *A*. *terreus*, thus pointing to a possible role of the rate of expression in *cadA* at least. On the other hand, Yin et al. (2017) investigated the transcriptome of two *A*. *niger* strains with different productivity during citric acid accumulation and found no significant differences in the expression of genes involved in its biosynthesis and further metabolism. This indicates the necessity for further studies in this area.

The only information concerning a possible wide domain control of citric and itaconic acid relate their overproduction to the protein methyltransferase LaeA (Niu et al. 2015), a protein associated with the conserved transcriptional regulatory velvet complex (Bayram et al. 2008). LaeA has been identified as an activator of secondary metabolite production, but was later also shown to control cellular development and extracellular enzyme formation (Bayram and Braus 2012; Karimi-Aghcheh et al. 2014). Several, but not all LaeA targets have been shown to occur as gene clusters. It would therefore be obvious to explain the effect of LaeA on itaconic acid accumulation on this basis. However, this would not account for the effect on citric acid accumulation: we have checked the genomic location of the genes for citric acid biosynthesis from glucose, and did not find any evidence for their clustering (Supplementary Table S4). Seven cases were found where two genes are in close vicinity, among which are the mitochondrial citrate synthase *citA* and the tricarboxylic acid transporter *ctpA*; the gene encoding cytosolic citrate synthase CitB and the one encoding the unknown Zn_2_Cys_6_ transcriptional activator that is part of the “itaconate cluster”(see above); and an aconitase with a putative mitochondrial tricarboxylic acid transporter. While we are unaware whether LaeA would specifically act on gene pairs, these data rather suggest that the effect of LaeA on citric acid and itaconic acid biosynthesis cannot be due to an activation of gene clusters synthesizing them. Unfortunately, no gene expression data have yet been published on citric and itaconic acid accumulation in *laeA* overexpressing and *laeA* knock-out mutants.

On the other hand, LaeA has been demonstrated to also act on signal transduction and this could be hypothesized to be a reason for its effect on acidogenesis. LaeA has been shown to be necessary for oxidative stress response in *A*. *fumigatus* (Fréalle et al. 2013). A role of LaeA in oxidative stress response would be in agreement with its essential role in fungal sporulation (Bayram and Braus 2012), its necessity for extracellular enzyme production (Karimi-Aghcheh et al. 2014), and requirement for secondary metabolite production (Németh et al. 2016). Since both citric and itaconic acid require high oxygen tensions for their accumulation, they are very likely under oxidative stress, although this has never been experimentally investigated so far. Further studies on the role of LaeA on the transcriptome of *A*. *niger* and *A*. *terreus* under acid-overproducing conditions may therefore contribute significantly to the understanding of these two processes.

## Concluding remarks

Although the fundamentals of the fermentation production of citric and itaconic acid have been established for a long while now, and (with the exception of two final steps for itaconic acid formation) the biochemical pathways involved are the same, few attempts have been made for mutual application of this knowledge. Based on the data discussed in this review, there is no need to study the production of citric or itaconic acid under conditions resulting in low titers because both can be produced at high molar yields (*Y*_p/s_ > 0.7) in their native producers, and there is no reason to assume that *A*. *niger* is better suited/adapted for the accumulation of organic acids than *A*. *terreus*. This particularly accounts for the attempts to produce itaconic acid heterologously in *A*. *niger*: while this may be attractive for reasons of industrial proprietary rights, patents, and regulations, it is still unclear if recombinant strains of *A*. *niger* would produce more itaconic acid than the industrial *A*. *terreus* strains currently in use, but the data presented in this review make this rather unlikely.

The only differences between citric acid and itaconic acid formation are the two additional steps encoded by *mttA* and *cadA.* The two encoded proteins appear to have orthologs in *A*. *niger* (and some *Penicillium*/*Talaromyces* spp. in the case of *cadA*), which suggests that they have a special evolutionary origin. Since they occur in cluster with two other genes (a Zn_2_Cys_6_ transcription factor and a MFS permease), one would expect that these two genes have a limited distribution, too. In contrast, they are present in a broad range of fungi. Most intriguingly, the *A*. *niger* orthologue of the Zn_2_Cys_6_ transcription factor also occurs in a gene cluster that encodes enzymes that could have the ability to form tensyuic acid, an alkyl-itaconic acid. The presence of a putative methylcitrate dehydratase (which has no close similarity to CadA), but no CadA in this cluster raises the question whether this methylcitrate dehydratase (and maybe others as well) could form itaconate. In addition, an investigation of the presence of these two clusters in other *Aspergillus* sps. or Eurotiales may illuminate their origin and evolution.

Understanding the regulation and compartmentation of citric acid and itaconic acid biosynthesis is still insufficient. This warrants more studies with gene deletion mutants that could answer—among others—the following questions: are both citric and itaconic acid regulated by the Zn_2_Cys_6_ transcription factor? Are MttA and CitB essential for itaconic acid formation (which would support/reject that hypothesis of a cytosolic biosynthesis)?

Summarizing, a wealth of information is now available about the biosynthesis of citric acid and itaconic acid, but deeper cell biology studies (regulation, compartmentation, energy balance) are still lacking. Such experiments must be performed under conditions of very high productivity because several aspects of cell physiology (compartmentation, gene expression, etc.) may adapt to the intensity of the acid-producing biosynthetic pathways. Finally, if a gene to be investigated is available in both *A*. *niger* and *A*. *terreus*, we recommend to perform the study in both fungi as the results are likely relevant to the accumulation of both acids.

## Electronic supplementary material

ESM 1 (PDF 4960 kb)
