# Supplementary material for: Citric acid and itaconic acid accumulation: variations of the same story?
Source: Appl Microbiol Biotechnol. 2019 Feb 13;103(7):2889–902. doi: 10.1007/s00253-018-09607-9 (PMC6447509; doi:10.1007/s00253-018-09607-9)
Supplement: Supplementary file 1 — (PDF 4960 kb) [file 253_2018_9607_MOESM1_ESM.pdf]

Supplementary Materials

Applied Microbiology and Biotechnology

Citric acid and itaconic acid accumulation: variations of the same story?

Levente Karaffa[1*] & Christian P. Kubicek[2]

[1]Department of Biochemical Engineering, Faculty of Science and Technology, University of Debrecen, H-4032, Egyetem tér 1, Debrecen, Hungary
[2]Institute of Chemical, Environmental & Bioscience Engineering, TU Wien, 1060, Getreidemarkt 9/166.5, Vienna, Austria

* Corresponding author
Tel.: +36 52 512 900 ext. 62488
E-mail: levente.karaffa@science.unideb.hu

Supplementary Table S1
Supplementary Table S2
Supplementary Table S3
Supplementary Table S4
Supplementary Table S5
Supplementary Table S6
Supplementary Table S7
Supplementary Table S8

Supplementary Table S1: Overview of citric acid fermentation technology data

Supplementary Table S1: Overview of citric acid fermentation technology data

		Medium components (g L^{-1})																													Fermentors														
References	Strain	Carbon source (g L^{-1})	Yield (g g^{-1} %)	$(NH_4)_2SO_4$	NH_4NO_3	$NH_4H_2PO_4$	Other N-source	$MgSO_4$	$MgSO_4$ 7H_2O	KH_2PO_4	K_2HPO_4	K_2SO_4	KCl	$NaNO_3$	$Fe_2(SO)_4 \cdot 24H_2O$	$FeSO_4 \cdot 7H_2O$	$ZnSO_4 \cdot 7H_2O$	$CuSO_4 \cdot 5H_2O$	NaCl	$CaCO_3$	$CaCl_2 \cdot 6H_2O$	$MnCl_2 \cdot 4H_2O$	MnSO4· 4H2O	Cu(II)	Zn(II)	Fe(III)	Mn(II)	HCl	Organic acid	Other media comp.	Fermentor/shaken flasks	Inoculation	Impellers	Working volume (L)	Air supply	Speed of stirring	DOT/redox level	pH	Temp (°C)	Fermentation time	Exhaust gas	Morphology	Pressure	Products	Comment
Wehmer patent (1893)	*Penicillium glaucum*	10-20 % sugar	N/A		small quantity			small quantity		small quantity																			2-5 % CA in media		large vessels (not described in patent)								ordinary temperature	8-14 days				CA, carbonic acid	
**US515033A**	*(Citromyces)*																																						(not controlled)					one unknown component	
Zahorsky patent (1913)	*Sterigmatocystis nigra*	5% sugar	N/A		small quantity			small quantity		small quantity																			15 % CA										10-30°C						
**US1066358A**	*(A. niger)*																																												
	1st niger fermentation																																												
Currie (1917)	*A. niger*	30 Cane sugar	N/A			2		0,2					0,2																		200 mL ERL			50 mL					28 °C						
		150 Saccharose	N/A		2,5				0,2	1						0,01												10 mL N/5 (0,02M)			200 mL ERL			50 mL					28 °C						
		50 Saccharose	N/A						0,2	1				2																	200 mL ERL			50 mL				1,7	28 °C	3-5 days				3 day Oxalic-; 5 days- CA	pH 1,4-1,6 critical
Media selection:		50 Saccharose	N/A						0,5	1			0,5	2		0,01															200 mL ERL			50 mL					28 °C						Czapek's solution
(based on Czapek's solution)		50 Saccharose	N/A						0,25	1			0,25	varying		0,01															200 mL ERL			50 mL					28 °C	7 days				CA, oxalic acid	best CA in 1,2 g NaNO3
		50 Saccharose	N/A			2		0,2					varying																		200 mL ERL			50 mL					28 °C	8 days				CA, oxalic acid	best CA depends on strain, but use 0,2 and 0,4 g KCl
		50 Saccharose	N/A						varying	1			0,25	2		0,01															200 mL ERL			50 mL					28 °C	5 days				CA, oxalic acid	between 0,1 and 0,2 g per liter MgSO4 optimal
		50 Saccharose	N/A						0,25	1			0,25	3		varying															200 mL ERL			50 mL					28 °C	7 days				CA, oxalic acid	0,01 g/L FeSO4X7H2O
In this paper best media:		125-150 Saccharose	around 16,7*		2,0-2,5				0,20-0,25	0,75-1,0																												3,4-3,5 (with HCl)							
Fernbach patent (1925)	*A. niger*	10-20 % sugar	N/A																																				10-40 °C						
**US1691966A**																																													
Scale up patent																																													
Josef Szücs patent (1928)	A.niger	Molasses	N/A																																				15-30°C					CA, without oxalic acid	
**US169186A**																																													
Franz Kanhause patent (1930)	A.niger	150 Saccharose	28		2			0,2		1																						fresh selected A.niger not yet cultivated			aeration				25°C	15 days				42 g/L Ca-citrate - 28% CA, remain 14 g/L saccharose	
**US1779001A**		150 Saccharose	46,6		2			0,2		1																						cultivated spores			aeration				25°C	15 days				70g/L Ca-citrate - 46,6% CA, remain 12 g/L sugar	
		150 Saccharified maize solution	69,3				4 g N2 from hydrolised glue sol.	0,2		0,4		1																				cultivated spores			aeration			6	25°C	15 days				104g/L Ca-citrate - 69,3%CA, remain 10 g/L sugar	
Doelger and Prescott (1934)	*A. niger TC67*	140 Sucrose	49		2,23				0,23	1																					250 mL ERL	10 days old spores		75 mL				3,0 (with HCl)	22-33 °C	9-12 days					
	isolated from rotted oak bark																																												
Tadeusz and Karol (1936)	*B. cinera*	2,5 % Ca-lactate + 1% CaCO3	8,3																													mycelium		100 mL					22 °C	12 days				8,3% of substrate used by mould to form	
		5 % Ca-lactate + 1% CaCO3	4,1																													mycelium		100 mL					22 °C	18 days				4,1% of substrate used by mould to form	
		5 % NH4-lactate + 3% CaCO3	4																													mycelium		100 mL					22 °C	21 days				4,0% of substrate used by mould to form	
		5 % NH4-lactate	0,8																													mycelium		100 mL					22 °C	18 days				0,8% of substrate used by mould to form	
		2,7 % EtOH + 4%CaCO3	5,95																													mycelium		100 mL					22 °C	18 days				5,95% of substrate used by mould to form	
Josef Szücs patent (1946)	A.niger	160 Refined sucrose	92		3,3				4,5	0,15			0,3								3,0 (x2H2O)							17,5 mL 1M		0,5 g/L dry milk		spores or fresh youg mycelium	rotary stirrer	2 L	75 mL/min	300 RPM		1,85	18-25°C	9 days				yield of 92% CA, absence of milk 70-75%	
**US2438136A**																																													
Karow et al (1947)	*A.wentii*	150 Sucrose	30,8				1 g urea		0,5	0,08			0,15				0,01						0,02								250 mL ERL	1 mL spore suspension		100 mL	2 L/h (by aerating tubed)	150 RPM shaker		2,0 (with HCl)	28 °C					23,7 g/L CA	
		150 Sucrose	25																																										
		150 Sucrose	31,8																																										
		150 Sucrose	40																																										
		150 Sucrose	35,8																																										
Shu and Johnson (1947)	*A.niger 72-4*	140 Domino sucrose	80		2,5				0,25	1														0,06 mg	0,25 mg	1,3 mg	<1 µg				500 mL cotton-plugged ERL			50 mL		270 RPM (1 inch rotary diameter)		3,1 (with HCl)	25°C	10 days					Mn-effect
Shu and Johnson (1948)	*A.niger 72-4*	68-145-264-312 Domino sucrose	46-64-62-55		2,5				0,25	1														0,06 mg	0,25 mg	1,3 mg	<1 µg				500 mL cotton-plugged ERL	1,5 mL spore suspension		50 mL		270 RPM (1 inch rotary diameter)			25°C	9 days				46-64-62-55 % CA (available sugar: 46-6	Products yield of CA on utilized sugar
vol40 No7					2,5																																								
Campbell et al (1950)	*A.niger 72-4*	150 Sucrose	16,7		2,8				0,3		0,6														5,0 µg	1,1 µg	above 0,4 µg/L CA production decrease											2							effects
																																													omitting Zn - 96% loss in CA production
																																													omitting Fe - 91% loss in CA production
																																													omitting Mn - 44% loss in CA production
																																													omitting Cu - 76% loss in CA production
																																													Omitting all 4 elements - 93% loss in CA production
Campbell et al (1953)	*A.niger 72-4*	150 Sucrose	65,8		2,8				0,3		0,6													0,5 mg/L	0,11 mg/L	0,04 mg/L	3 ppb											2	25°C (best result)	10 days				10g CA	
Steel et al (1955)	*A.niger N.R.C. A1-233*	128,16 Sucrose (molasses)	71,1																																sintered glass aerotation disk										
increase scale	small fermenters																													lard oil (antifoam)	2,5 L glass with mechanical foam breaker	200000 pellets/L			500 mL/min			initial pH=7,0	31 °C controlled	117 h		pellet		CA 71,1% conversion in Chatham1953 molasses 117h	
	large fermenters																													lard oil (antifoam)	36-40 L 304 and 316 stainless steel G and H por	200000 pellets/L			6400 mL/min			initial pH=7,0	31 °C controlled			pellet		CA 71,1% conversion in Chatham1953 molasses 117h	
Clark and Lentz (1961)	*A.niger N.R.C. A1-233*	120 Chatham 1957 beet molasses	76																											0,4 g K-ferrocyanide	36 L glass tower fermenter	29 °C 10^6 spores 20-24h on rotary shaker		20 L	2500 mL/min			initial pH=7,0	31 °C controlled	140h		pellet	1,7 atm	avegare conversion 76% (sucrose to CA)	
		120 Chatham 1958 beet molasses																												0,7 g K-ferrocyanide				20 L	2500 mL/min			initial pH=6,5	31 °C controlled	140h		pellet	1,7 atm	avegare conversion 76% (sucrose to CA)	
Clark (1962)	*A.niger N.R.C. A1-233*	120 Chatham beet molasses	N/A																											15 ppm ferrocyanide	Pyrex tower fermenter	29 °C 10^6 spores 24h on rotary shaker			300 mL/min/L				31 °C controlled	140h			1 atm		0,5 mm pellets
Svenska patent (1964) abstract	A. niger	150 Sugar beat molasses	N/A																											0,2% ferrocyanide					29,5-29,8 DEG C			below 3,5				pellet	subatmospheric	15 % CA (free from oxalic acid)	
**Br. Pat., 951,629**																																													
Clark et al. (1966)	*A.niger N.R.C. A1-233*	120 Sugar beat molasses (pH=6)	77,5																								0-1000 ppb			20 ppm ferrocyanide	2,5 L glass tower fermenter	29 °C 10^6 spores 18-24h on rotary shaker			700 mL/min				31 °C ±1 controlled	140 h		pellet/filamentous		1ppm Mn(II) - 9 % CA (140h) 93g/L	2 ppb Mn(II) - slightly filamentous morphology
		120	78,3																																									94 g/L CA	
		120	76,7																																									92 g/L CA	
		120	77,5																																									93 g/L CA	
		120	66,7																																									80 g/L CA	
		120	50																																									60 g/L CA	
		120	33,3																																									40g/L CA	
		120	16,7																																									20 g/L CA	
		120	17,5																																									21 g/L CA	
Ahmed et al. (1972)	*A.niger 72-4; A-I-233*	140 Sucrose	89,8		2,5				0,25		1						2,2									$FeCl_3 \cdot 6H_2O$: 6,27 mg					500 ml wide-mouthed conical flasks							3,1	28 °C						
Hamissa et al. (1977)	*A.niger NRRL 599*	Molasses 30%	59,73		0,20%																									methanol/ethanol (2/2,5/3 v/v%)	fermentation trays (Alu, PE, stainless steel)							5,6	30 °C						
																														$K_4Fe(CN)_6$ (0,03/0,06/0,09/0,12/0,15 g%)															
Kristiansen et al. (1978)	*A. foetidus IMI 5954*	130 Sucrose	6,53		1 kg/m3				0,5 kg/m3	1 kg/m3					0,5 g/m3		0,25 g/m3	0,06													10 liter glass vessel		six-bladed turbine of conventional design	8	0,5 l/l/min	300	-	4	28 °C						
		140	7,86		2,5				0,5	1					0,375		0,25	0,06																8	0,5	300	-	4	28 °C						
		135	15,18		2,5				0,5	1					0,375		0,25	0,06																8	0,6	300	1	5,4	28 °C						
		65+60 (fed-batch)	7,84*		1,5				0,5	2					0,1		0,1	0,06																8	0,6	350	0,21	6,1	32 °C						
		130	0,85		1,5				0,5	2					0,1		0,1	0,06																8,5	0,7	350	0,21	4	32 °C						
		140	15		1,5				0,5	2					0,1		0,1	0,06																8,5	0,7	350	0,21	6,5	32 °C						
		135	12,6		1,5				0,5	2					0,1		0,1	0,06																8,5	0,7	350	0,21	6,5	32 °C						
		140	13,14		1,5				0,5	2					0,1		0,1	0,06																8,5	0,7	350	0,21	3	32 °C						
Nubel, R. et al. patent (1979)	*Candida lipolytica ATCC 20,228*	C_{14}-C_{16} n-paraffin/n-hexadecan: 180 g	50-75				carbamide: 4,7 g			0,375 g																				tiamin-HCl: 0,001 g	"large" fermenter				~30 m3 air/m3 liq./h	1725 rpm		2-3	26 °C						
		C_{14}-C_{16} n-paraffin: 180 g					carbamide: 4,7 g		0,1	0,75																				tiamin-HCl: 0,01 g					~30 m3 air/m3 liq./h	1725 rpm		2-2,5	25 °C						
**US4155811**																																													
Roehr et al (1981)	*A. niger B60/B3 and B60/B5b*	226 Sucrose	34,5						1,1	0,15							0,0015		0,15		0,55 (2 H2O)										50 l Pilot fermentor (Bioengineering AG, Wald, Switzerland)		Flat-blade impeller	35	0,5 vvm	1650 rpm		3	30 °C			hyphae/pellet			
Heinrich et al. (1982)	*A. niger ATCC 9142*	150 Sucrose	14*		0,5 g				0,3	0,2						0,0005	0,0011																			500-1000 RPM		4							
Berovic et al. (1982)	*A. niger A60 (NRRL 2270)*	Deluted beat molasses (12% sugar c.)	62,5*																												5 L Bioengineering AG lab. Fermenter	4x10^7 conidia/L			1 vvm	600 RPM		4	30 °C						
M.Hossain et al. (1984)	*A. niger MH 15-15*	140 Sucrose	34				2 g NaNO3		0,5	2					0,0001		0,0001	0,00006													Microferm Fermenter			5 L	3-5 L/min	200-300 RPM		6,5	30						
		140 Fructose	13																																										
		140 Lactose	9																																										
		140 Galactose	7																																										
		140 Glucose	0																																										
Eikmeier et al. (1984)	*A. niger ATCC 11414*	250 Sucrose	50		0-0,4				0,3	0,2						0,0001	0,0025														Fernbach flasks	0,4X10^8 spore/mL		300 ml		125 RPM			30						
																															500 ml														
Kirimura (1988)	*A. niger WU-2223L*	120 Sucrose	83*	3					0,5	1	1																				Sakaguchi Flasks (500 ml)			60 mL		120 RPM		3	30						
Träger et al. (1989)	A. niger RRL 12-16/1-2	40 Glucose monohydrate	0		1				0,25	1										13											Stirred tank, laboratory airlift fermentor, 260 L Airlift fermentor			Stirred: 10 L	Stirred: 5 L/min	Stirred: 600 rpm		6,5	30						
	gluconic acid production	100 Glu-monohydrate (260 L airlift exp.)																																Airlift: 4 L	Airlift: 4 L/min										
																																			260 L Airlift: 35 L/min										
Aravantinos-Zafiris et al (1994)	*A. niger NRRL 567 & 364 & 599*	56.0 g kg-' with a	730 g kg^{-1*}																																			3.0-6.2	30 °C						
		glucose : fructose : sucrose																																										30 g of citric acid per kg	
Arisan-Atac et al (1996)	*A. niger ATCC 114 14(mutant of it*	80 Glucose	66,3																																										
	ATCC11414	50	58,6																																										
		25	22,8																																										
		10	15,2																																										
BERCOVITZ et al (1990)	*14 strains of Aspergillus species*		N/A																																										
Friesen et al (1991)	*Aspergillus or Candida*	Glucose	N/A																																				60 °C						
Garg and Hang (1995)	*A. niger, Rhizophos oryzee*		N/A																																										
Gupta and Sharma (1995)	*A. niger GS-III (mutant)*	Sugarcane molasses (120 total sugar)	75*		0,25%				0,03%		0. l %											0.0-4.0																4.0-4.5	room teTmperature 30 C					CA 90 KgM-3	
Hang (1998)	*A. niger NRRL 2001, 2270, 328, 59*	Glucose	over 50																																				23, 30,37°C	0, 24, 48, 72, 96, 120, 144 h				CA	
JAKLITSC et al (1991)	*A. niger B60*	140 Sucrose	37*		5				0,25	0,5							1.1 mg/l	0.2 mg/l								3.8 mg FeCl												3,5	30°C	up to 6 day				CA	
Jianlong Wang (1998)	*A. niger W1-2*	Beet molasses (100 total reducig sugar)	38*						0,25	1																					200							4	30°C					CA	
Khare et al (1995)	*A. niger NRRL 330 & A. terreus NCIM 653*	Okara (soy-residue),	25,61																																			8,3	30°C					5.10 g citric acid/IO0 grams dry solids	
Kirimura et al (1992)	*A. niger WU-2223L*	120 Glucose	56	3					500mg/l	1	1												14 mg/l															3	30°C					CA	
Kontopidis et al (1995)	*A. niger SZ 1*	150 D-glucose	87,5		0,5				0,25	0,1			0.428 g/l			0,0037	0,02				0.128g/1																		29°C	190 h				CITRATE TRANSPORT	
Krzystek et al (1996)	*A. niger IH II*	100 Sucrose	96																																		were not controlled		32 +0.1 °C					CA	
Kubicek Pranz et al (1990)	*A. niger ATCC 11 414*	Sucrose	N/A	2,5					1,1	0,15							0,0015		0,15																			3	30°C					CA	
Lu et al (1997)	*A. niger Yang No. 2*	Kumara (Ipomoea batatas)	maximum 60																																				29- 30 C					0.82 g citrate kg-1	
Lu et al (1998)	*A. niger Yang No. 2*	Kumara (Ipomoea batatas)	55,1																																										
McIntyre and McNeil (1997)	*A. niger (A60)*	140 Sucrose	62	0.1 x 10-3					0,5	2						0.1 x 10-3	1 x 10-3																					1,8	30°C.	43-74 h				CA	
Navaratnam et al (1998)	*A. niger*	50 Glucose	9,2*	0,5					0,1	0,5							0.1 x 10-3	0.06 x 10-3																						120 h				CA	
Pallares et al (1996)	*A. niger CBS 733-88,*	100 Glucose	20,6*		1,14				0,3	0,261						0.1 mg/1	0.25 mg/1																	700ml				4	30°C.					CA	reducing the fermentation time from 14 to 6 days
Papagianni et al (1994)	*A. niger PM 1*	150 D-glucose	80		2,5				0,25	0,1			0.428 g/l			0.75 mg/l	4.5 mg/l				0.252 g/l													220 dm 3					28 °C						
Papagianni et al (1998)	*A. niger (PM1)*	150 D-glucose	80,7		2,5				0,25	0,1			0,428			0.75 mg/l	4.5 mg/l				0,252																		30						
		150 D-glucose	81,7																																										
Papagianni et al (1999)	*A. niger PM1*	150 D-glucose	83,3*		2,5				0,25	0,1			0,43			0,75x10-3	4,5x10-3				0,25																								
Papagianni et al (1999_1)	*A. niger PM1*	150 D-glucose	29* (batch 200 RPM)																																										
			80,7* (batch 400 RPM)																																										
Papagianni et al (1999_2)	*A. niger PM1*	150 D-glucose	80,7*																																										
		100 D-glucose	87,5*																																										
		60 D-glucose	45*																																										
Papagianni et al (1999_3)	*A. niger PM1*	156,7 D-glucose	69,8*																																										
Roukas (1991)	*A. niger ATCC 9142*	Beet Molasses	55																																			6,5	30°C					CA	
Roukas (1998)	*A. niger ATCC 9142*	Carob tree (Ceratonia siliqua) 100 initial sugar	73																																										
		150	56																																										
		200	50																																										
		250	41																																										
Sarangbina& Watanapokasin (1999)	*Mutant of A. niger Yang no. 2*	Yam bean starch	58-74		2				250 mg/l	10													14 mg/l															4,25	30°C					CA	
Shankaranand and Lonsane (1993)	*A. niger CFTRI 30*	Sugarcane-pressmud	70 and 80																															8 L											
Shankaranand and Lonsane (1994)	*A. niger CFTRI 30*	Coffee husk	82																																										
Tran et al (1998)	*A. foetidus ACM 3996 and 3 A. ni*	Wet pineapple waste	74																																										
Papagianni et al. (2005)	*A. niger PM1*	150 D-Glucose	73	2,5					0,5	2						0,0001	0,0001	0,00006													New Brunswick Scientific BIOFLO 410 model		three disk turbine impellers, 8 cm in diameter, with six flat blades	10	1vvm	600		2,1	28			clumps			
				0,5																																									
				1																																									
				2																																									
Dai et al. (2003)	*A. niger ATCC 11414*		23,6* (10ppb Mn(II))		3,1																																								
		140 Glucose	0* (1000ppb Mn(II))						2,2	0,15							0,0066	0,0001	0,15			10ppb/1000ppb									baffled flasks			250/1000mL		250rpm		2	30			pellet/filamentous growth			
Papagianni and Mattey (2006)	*A. niger PM1*	150 D-glucose	66,7	2,5					0,5	2						0,0001	0,0001	0,00006													New Brunswick Scientific BIOFLO 110		two 6-bladded Rushton-type impellers (52 mm)	3	1vvm	400		2,1	28			morphology analysis			
	A. niger PM1		73,3																																										
Papagianni (2006)		150 D-glucose	6,67	2,5					0,5	2						0,0001	0,0001	0,00006																											
			6,67							0,5/1/2,5												10/20/30 ug/L									New Brunswick Scientific BIOFLO 110		two 6-bladded Rushton-type impellers (52 mm)	3	1vvm	400		2,1	28			morphology analysis			
																																										clump/pellet			
Müge et al. (2002)	*A. niger NRRL2270*	100 Sucrose	40		0,025																																								
									0,25	1						0,0013	0,00025	0,00006															Shaken flasks	150mL	3vvm	100rpm		4,5	30						
Bizujkojc et al. (2003)	*A. niger BIO4F*	100 sucrose /glucose/fructose		2					0,15	0,15																																			
		air flow, overpressure	87,42 (best)																																										
																																	B-Braun; tank reactor and air lift, shaken flasks	16,51-reactor	1-20L/min	max 350rpm	DO>15%	2,5	30				atmospheric and overpressure:0,25MPa		
Adham (2002)	*A. niger A20*	Beet molasses+tap water	17* (surface control)																																										
				0,05																													Erlenmeyer flasks	50mL				5-5,7	30						
Papagianni et al. (2004)	*A. niger*	Glucose	N/A																																										
		150/130/ 886 70/ 44/ 17/5		2,5					0,25	0,1			0,43			0,00075	0,0045	0,43			0,25										BIOSTAT, ED-ES10, stirred tank		three disc turbine impellers, each with six flat blades	800mL	1vvm	400rpm		2,1	28						
Pinacci et al. (2002)	*A. niger*		60-75																																										
Recovery of CA from broth																															Electrodyalisis membrane								25-35						
Rivas et al. (2008)	*A. niger CECT-2090*	Orange peel hydrolysate	74 (highest)																	20																			30						
		400 g+ methanol 0/20/40/60/80 mL/kg																													flasks														
Rywiska (2011)	*Y. lipolitica AWG7*	150 glycerol	59 (D=0,009h-1)				3 g/L NH4Cl		1	0,2																				1 g/L yeast extr.	5L Biostat B-Plus Sartorius, Germany			2 L	0,36 VVM	600 RPM		maintanied 5,5	30						
chemostat			61 (D=0,015h-1)																																										
D=0,009; 0,015; 0,021; 0,024; 0,031 h^-1			67 (D=0,021h-1)																																										
			64 (D=0,024h-1)																																										
			61 (D=0,031h-1)																																										

Supplementary Table S2: Overview of itaconic acid fermentation technology data

		Medium components (g L^{-1})																																											
References	Strain	Carbon source (g L^{-1})	Yield (g g^{-1} %)	$(NH_4)_2SO_4$	NH_4NO_3	$NH_4H_2PO_4$	Other N-source	$MgSO_4$	$MgSO_4$ 7H_2O	KH_2PO_4	K_2HPO_4	K2SO4	KCl	$NaNO_3$	$Fe_2(SO)_4$ · 24H_2O	$FeSO_4$ · 7 H_2O	$ZnSO_4$ ·7H_2O	$CuSO_4$ · 5H_2O	NaCl	$CaSO_4$	$CaCl_2$· 6H_2O	$MnCl_2$· 4H_2O	MnSO4· 4H_2O	Cu(II)	Zn(II)	Fe(III)	Mn(II)	HCl	Organic acid	Other media comp.	Fermentor/shaken flasks	Inoculation	Impellers	working volume	air supply	speed of stirring	DO (%)	pH	Temp (°C)	Fermentation time	exhaust gas	morphology	Pressure	Products	Comment
Calam et al. 1939	L.S.H.T.M. Cat. No. Am. 1	50 D-glucose	9						0,5	1			0,5	2		0,01															1L flask	spores		350 ml		not shaked		5	24	7 days				0,44 g/L IA	
Moyer & Coghill 1945	NRRL 265	250 D-glucose	29		2				0,25				0,05				0,044													4 ml CSL, 8 mg/L ferreous-tartarate	200 ml Erlenmeyer	spores		60 ml		not shaked		1,80-1,90	27	10-12 days				72,5 g/L IA	
Lockwood and Ward 1945	NRRL 1960	165 D-glucose monohydrate	54		2,5				4,4								$4,4*10^{-3}$		0,4											4 ml CSL,	pan	spores		12 L	5 L/min			2	30-32	12 days	1% CO_2, 20,5 % O_2			50 g/L IA	
Kane patent 1945<br>**US2385283**	N/A	98 sucrose	25											15																0,5 g/L Na-aliginate, combined phos phorus and potash and trace elements	Stirred tank reactor	spores	propeller	N/A	N/A			N/A	34-38	7 days				24 g/L IA	
Lockwood and Nelson 1946	NRRL 1960	74 D-glucose	34		2,5				0,75	0,6																				1,5 ml CSL	N/A	preculture from shake flasks		N/A	N/A	N/A		keep 1,80	30	12 days		Inoculum: pellets 2mm diameter		25 g/L IA	
Nelson et al. 1952	NRRL 1960	59-88 D-glucose	46	2,67					5																						20L Sirred tank reactor	preculture from shake flasks	six-vaned agitator	15 L	0,025 vvm	100 rpm		keep 1,80-2,00	34	258 hours		Inoculum: pellets 1-2 mm diameter	+0,1 bar	27,3 g/L IA	15 mg/L Fe^{2+} already decreased the yield
Pfeifer et al. 1952	NRRL 1960	66 D-glucose monohydrate	60	2,7					0,8																					1,8 g/L CSL	2270L Stirred tank reactor	10% (v/v) inoculation from mycelia	turbine	N/A	0,25 vvm	77-230 rpm		4,5	34-40	80 hours			+0,1 bar	33 g/L IA	Fe is toxic for fermentation
Larsen and Eimhjellen 1955a		50 D-glucose	46	3					0,5	0,1										3											500 ml Erlenmeyer	1-2 sq.cm. of a spore mat		100 ml		180 rpm		5,5		120 hours	0,34 g/g CO_2			23,2 g/L IA	
		50 D-glucose	0																	3											500 ml Erlenmeyer	1-2 sq.cm. of a spore mat		100 ml		180 rpm		keep 6,00 with $CaCO_3$		120 hours	0,50 g/g CO_2			0 g/L IA	
Larsen and Eimhjellen 1955b	NRRL 1960	50 Sucrose	57	3					0,5	0,1										3											500 ml Erlenmeyer	1-2 sq.cm. of a spore mat		100 ml		180 rpm		5,5	30	50-60 hours				28,2 g/L IA	
		50 D-mannose	32																												500 ml Erlenmeyer			100 ml		180 rpm				50-60 hours				16 g/L IA	
		50 D-xylose	31																												500 ml Erlenmeyer			100 ml		180 rpm				50-60 hours				15,5 g/L IA	
Nubel patent 1962<br>**US3044941**	NRRL 1960	150 beet molasses	57				molasses		3								1 (only $ZnSO_4$)	0,01									Limited				Stirred tank reactor	25% (v/v) inoculation from mycelia	propeller	2 L	0,25-0,5 vvm	N/A		5,1 produced IA neutralized by KOH, NaOH, or NH4OH (age 18-48h)	35-42	60-80 hours				85 g/L IA	molasses, treated with activated carbon-ion exchange or Potassium-ferrocyanide
Batti patent 1963<br>**US3078217**	NRRL 1960	130 Sucrose	58*				2 g/L NH_4HCO_3		1		0,14									12 g/L $CaSO_4$*2 H_2O				$3*10^{-3}$	$3*10^{-3}$		Limited				Stirred tank reactor	spores	N/A	4 L	1,25 vvm at first 24h, 1,50 vvm after			3,00-4,00	32-35	160-200 hours				79,5 g/L IA	Fe(III) decreases yield already at 1 mg/L, ion-exchange for carbon source
Batti patent 1964<br>**US3162582**	N/A	150 sucrose	N/A																												N/A	N/A	N/A	N/A	N/A	N/A		3,00-5,00	N/A	7 days				When the IA hasn't neutralized succinic and itartartaric acid appeared	Start the fermentation with "high" pH (ex. 5,10) leads to 15% by-product ratio.
Kobayashi 1966	ATCC 10020	120 D-glucose	60	3					0,5	0,1										3 g/L $CaSO_4$*2 H_2O											20L Sirred tank reactor	N/A	disc type with curved blades, 15 cm diameter	N/A	N/A	500 rpm		N/A	35-40	N/A				N/A	
Kobayashi patent 1975<br>**US3873425**	ATCC 10020	70 raw cane sugar	71		3			1		0,2								$4*10^{-3}$ anhydrous CuSO4													20L Sirred tank reactor	5% (v/v) inoculation from mycelia	disc type with curved blades, 15 cm diameter	8 L	0,1 vvm	460 rpm		2,2	35	N/A				36,38 g/L IA	continuous, feeding after 40 hours of fermentation. Feeding rate: 500 ml/h
Kobayashi et al. 1977	ATCC 10020	100 D-glucose	55		3				2																					2 g/L CSL	500 ml Erlenmeyer	N/A		120 ml		N/A		N/A	36	5 days				54,40 g/L IA	
		100 D-Mannose	51																																					4,5 days				46,50 g/L IA	
		100 D-galactose	8																																					7,8 days				2 g/L IA	
		100 D-xylose	49																																					5,8 days				45,90 g/L IA	
		100 L-arabinose	34																																					7,8 days				29,50 g/L IA	
Rychtera and Wase 1981	NRRL 1960	50 D-glucose	64	3,3					3,3	0,088								$4*10^{-3}$												0,8 g/L itaconic acid	Stirred pipe reactor	7% (v/v) inoculation from mycelia	flat vertically mounted blade (8,5 * 3,5 cm)	3 L	0,2 vvm	160 rpm		various, best: 3,10	36	160 hours		Pellet diameters increase with pH (3,10→1mm, 3,50→5 mm), bigger pellet is unfavourable		32 g/L IA	batch
		50 D-glucose	26																																									13 g/L IA	continuous fermentation
Kautola et al. 1985	NRRL 1960	55 D-glucose	55	3,3					0,8	0,088								$4*10^{-3}$													14L Stirred tank reactor	10% (v/v) inoculation from mycelia	N/A	2 L	0,05 vvm	N/A	min 25	3,1	36	120 hours				30 g/L IA	
		67 D-xylose	45																												14L Stirred tank reactor			10 L						144 hours				30 g/L IA	
		60 D-xylose	12/batch																												250 ml Erlenmeyer			100 ml						120 hours				13,80 g/L IA	repeated batch fermentations with immobilized cells
		60 D-glucose	18/day																												air lift reactor			N/A	N/A		N/A			2 weeks with 9-10 hours residence time				11 g/L IA	continuous fermentation with immobilized cells
		60 D-xylose	8/day																												air lift reactor									2 weeks with 9-10 hours residence time				5 g/L IA	continuous fermentation with immobilized cells
Ju et al. 1986	NRRL 1960	50 D-glucose	49*	3,3					0,8		0,088							$4*10^{-3}$												1 g/L itaconic acid	Porous disk bioreactor	Immobilization of the mycelia on disks	disks	1,5 L	0,07 vvm	8 rpm		3	36	30 days				24,50 g/L IA	batch
		50 D-glucose	36*																																									18,40 g/L IA	5 days batch then change to continuous operation
Kokufuta et al. 1988	ATCC 10020	80 D-glucose	38		3				2																					2 g/L CSL	500 ml Erlenmeyer	10% (v/v) inoculation from mycelia		100 ml		120 rpm		3,8	30	88 hours				30,40 g/L IA	flocculation mycelia with potassium poly(vinyl alcohol) sulfate and poly(diallyldimethylammonium chloride) method gives lower IA yields but extremely decreases viscosity
Kautola et al. 1989	TKK-200-5-3	100 sucrose	18/batch		2,75			0,95										$4*10^{-3}$													250 ml Erlenmeyer	inoculate polyurethane cubes with spores (immobilization) and transfer to new flasks		100 ml		200 rpm		various, best: 3,00	35	14 days				18 g/L IA/batch	repeated batch fermentation with free and immobilized cells
Kautola et al. 1990a	TKK-200-5-3	90 D-glucose	28/batch*		2,5			0,95										$4*10^{-3}$													130ml air lift column reactor	inoculate polyurethane cubes with spores (immobilization) and transfer to fermenter		95 ml	1,15 vvm			3	36	178 hours				26 g/L IA	repeated batch with immobilized cells,lack of the $CuSO_4$ gave better yields
		135 sucrose	12/batch*																																									15,80 g/L IA	repeated batch with immobilized cells
Kautola et al. 1990b	TKK-200-5-3	various D-xylose, best:174	2/batch*		various, best: 1,60			0,95										$4*10^{-3}$													250 ml Erlenmeyer	inoculate polyurethane cubes with spores (immobilization) and transfer to flask/fermenter		100 ml		200 rpm		various, best: 2,50		14 days (change medium at every 3rd day				1,6 g/L IA	Repeated batch fermentation with immobilized cells
		various D-xylose, best:50	12*		1,6			0,95										$4*10^{-3}$													130ml air lift column reactor	inoculate polyurethane cubes with spores (immobilization) and transfer to flask/fermenter		95 ml	various, best: 0,6 vvm			2,5	36	160h				8,10 g/L IA	continuous fermentation with immobilized cells
		60 D-xylose	4*																												700ml air lift column reactor			620 ml	0,8 vvm			3	36	110h				2,54 g/L IA	batch with immobilized cells
		60 D-xylose	1*																												14L air lift reactor			9 L	1,1 vvm			3	36	110h				0,30 g/L IA	batch with immobilized cells
Kautola et al. 1991	NRRL 1960	various D-glucose, best:150	34*		4			various, best: 3,00										CuSO4 $19,6*10^{-3}$			$CaCl_2$ 6,5										250 ml Erlenmeyer	inoculate polyurethane cubes with spores (immobilization) and transfer to flask/fermenter		100 ml		200 rpm		3,1	36	14 days				51 g/L IA	repeated batch with immobilized cells
Vassilev et al. 1992	NRRL 1960	various D-glucose, best:150	4*		various, best: 4			0,95										$4*10^{-3}$													250 ml Erlenmeyer	inoculate polyurethane cubes with spores (immobilization) and transfer to flask/fermenter		100 ml		200 rpm		3,5	36	14 days/batch				7 g/L IA	repeated batch with immobilized cells
		various D-glucose, best:90	29*		2,5			0,95										$4*10^{-3}$													130ml air lift column reactor	inoculate polyurethane cubes with spores (immobilization) and transfer to flask/fermenter		95 ml	1,10 vvm			3		190 hours				26 g/L IA	continuous fermentation with immobilized cells
Park et al. 1993	IFO-6365	130 D-glucose	46		5				2																					1,8 g/L CSL	5L Stirred tank reactor	10% (v/v) inoculation from mycelia	6 cm diameter	2 L	0,5 vvm	200-400 rpm	min 20	3	40	160 hours				52 g/L IA	Do not increase impelled speed above 94 cm/sec, DO 20 % is enough to reach best IA yield
Okabe et al. 1993	IFO-6365	130 D-glucose	48		5				2																					1,8 g/L CSL	3L air lift reactor	10% (v/v) inoculation from mycelia		2 L	2 vvm		50	3	40	8 days		between pellet and pulp was the best to produce IA		63 g/L IA	Free cells give better IA production than immobilized ones
Park et al. 1994	IFO-6365	130 D-glucose	48		5				2																					1,8 g/L CSL	2L air lift reactor	10% (v/v) inoculation from mycelia		1,5 L	2 vvm		keep 20	3	40	5 days				48 g/L IA	
Yahiro et al. 1995	TN-484	140 D-glucose	52		2,9				1,8																					1,2 g/L CSL	2,5L air lift	spores		N/A	2 vvm		keep 20	2,9	37	6 days				72,5 g/L IA	
Gyamerah et al. 1995a	NRRL 1960	100 sucrose	55	3					0,5	0,1						0,06 mg/L				23 mg/L $CaSO_4$*2 H_2O					$1*10^{-3}$						500 ml Erlenmeyer	spores: 2-6*10^7 spores/ml		100 ml		160 rpm		no adjustment	30	192 hours		IA production is good when pellets are small and frayed (0,1-05 mm diameter). If the pellets are compact or too big the production is not good		55 g/L IA	Presence of Ca^{2+} Zn^{2+} Fe^{2+} are adequate to earn good IA yields. Morphology has to be small frayed pellets
Gyamerah et al. 1995b	NRRL 1960	100 D-glucose	52	3					0,5	0,1										3											500 ml Erlenmeyer	spores: 2-6*10^7 spores/ml		100 ml		160 rpm		6	30	240 hours				51,64 g/L IA	3 min stop of agitation caused 13 % decrease of IA prod.
																																													10 min stop of agitation caused 41 % decrease of IA prod. IA production interrupted and started again after 48-hours
Yahiro et al. 1997	TN-484	140 D-glucose	46*		2,9				1,8																					1,2 g/L CSL	3L air lift reactor	10% (v/v) inoculation from mycelia		2 L	2 vvm		dropped to 0 at 2nd and 3rd day of the cultivation, otherwise 90%	3	30	6 days				65 g/L IA	
		140 corn starch	58*																																0,5 vvm									58 g/L IA	
Petruccioli et al. 1999	NRRL 1960	60 D-glucose	43	2,36					2,1	0,11						$5,5*10^{-3}$	$1,3*10^{-3}$	$0,2*10^{-3}$	$74*10^{-3}$		0,13 g/L $CaCl_2$ *2 H_2O	0,7 mg/L $MnCl_2$									3L Stirred tank reactor	10% (v/v) inoculation from mycelia	2 six bladed Rushton 45 mm diameter	2L	1 vvm	500 rpm		3,4	35	144 hours				17,80 g/L IA	
		60 corn starch hydrolisate	33																																									18,20 g/L IA	
Riscaldati et al. 2000	NRRL 1960	100 D-glucose	54	2,36					2,1	0,11						$5,5*10^{-3}$	$1,3*10^{-3}$	$0,2*10^{-3}$	$74*10^{-3}$		0,13 g/L $CaCl_2$ *2 H_2O	0,7 mg/L $MnCl_2$									15L Stirred tank reactor	10% (v/v) inoculation from mycelia	two turbines with 6 flat blades (diameter 75mm)	9 L	0,4 vvm	320 rpm	about 20	initial: 3,40 let it fall to 2,40 naturally then keep it	35	10 days				57,20 g/L IA	
Reddy and Singh 2002	N45	120 corn starch	40		4			0,095										40 mg/L $CuSO_4$													250 ml Erlenmeyer	5% (v/v) inoculation from mycelia		100 ml		120 rpm		3	34	6 days				46 g/L IA	
		90 apple	37																																									29 g/L IA	
		90 banana	35																																									31 g/L IA	
Iqbal and Saeed 2005	NRRL 1960	90 D-glucose	26*		2			0,095										4 mg/L $CuSO_4$													250 ml Erlenmeyer	inoculate papaya cubes with spores (immobilization) and transfer to flask		100 ml		150 rpm		3	35	7 days				21,78 g/L IA	repeated batch with immobilized cells
Dwiarti et al. 2007	TN-484	140 sago starch	36		2,9				1,2																					1,8 g/L CSL	3L Stirred tank reactor	10% (v/v) inoculation from mycelia	N/A	2 L	0,5 vvm	290-310 rpm	3	2	40	6 days				48,20 g/L IA	
Tevž et al. 2010	A729	100 D-glucose	30*	2,36					2,08	0,11							$1,3*10^{-3}$		$74*10^{-3}$		0,13 g/L $CaCl_2$ *2 H_2O										5L Stirred tank reactor	10% (v/v) inoculation from mycelia	N/A	N/A	1 vvm	500 rpm		3,4	35	120 hours				30 g/L IA	ATCC 20542 has been transformed with modified *A. niger pfkA* gene.
Kuenz et al. 2012	DSMZ 23081	180 D-glucose	58		3				1								$8*10^{-3}$	15 mg/L $CuSO_4$ * 7 H_2O,			5 g/L $CaCl_2$ *2 H_2O					1,67 mg/L $FeCl_3$ * 6 H_2O,					250 ml Erlenmeyer	spores: 1*10^6 spore/ml		100 ml		120 rpm		3,1	33	10 days				70 g/L IA	IA production was decreased if the flasks were not shaked during sampling
		180 D-glucose	62																												1,5L Stirred tank reactor	spores: 1*10^6 spore/ml	magnetic stirrer	1 L	N/A	470 rpm	keep 20	3,1	33	7,8 days				86,20 g/L IA	
		150 D-glucose	62																												15L Stirred tank reactor	spores: 1*10^6 spore/ml	Rushton	10 L	N/A	350 rpm	keep 20	3,1	33	10 days				85 g/L IA	
Vassilev et al. 2012	CECT 20365	100 D-glucose	30*		1,5				0,1	0,5								5 mg/L $CuSO_4$												1-3 g/l HABO (hydroxyapatite of animal bone origin)	250 ml Erlenmeyer	inoculate polyurethane cubes with spores (immobilization) and transfer to flask		100 ml		200 rpm		4,5	37	120h				30,20 g/L IA	
		100 glycerol	27*																																									26,90 g/L IA	
Shin et al. 2013	R 104	132 sucrose	72	3				0,5		0,5						$0,2*10^{-3}$	$8,8*10^{-3}$	$0,4*10^{-3}$									0,1 mg/L $MnCl_2$ * 4 H_2O,			2 g/L Yeast extract, 0,005 mg/L Na2MoO4,	50L Stirred tank reactor	5% (v/v) inoculation from mycelia	2 turbine impellers with 6 blades/each 133 mm diameter	35 L	0,5 vvm	180 rpm	keep 20	2,1	37	132 hours				50 g/L IA	
Gao et al. 2014	FMME033	80 D-glucose	24	4					1	0,05						$40*10^{-3}$	$50*10^{-3}$													50 mg/L CSL	500 ml Erlenmeyer	spores: 1*10^9 spore/ml		100 ml		200 rpm		3,1	35	N/A		dispersed mycelia		7,1 g/L IA	
		80 D-glucose	50																												500 ml Erlenmeyer	spores: 1*10^8 spore/ml		100 ml		200 rpm				N/A		clump		29,4 g/L IA	
		80 D-glucose	38																												750 ml baffled flask	spores: 1*10^8 spore/ml		75 ml		200 rpm				N/A		pellet		16,40 g/L IA	
		80 D-glucose	50																												7L Stirred tank reactor	10% (v/v) inoculation from mycelia	N/A	4 L	0,8 vvm	300 rpm	N/A			48 hours		clumo		40 g/L IA	
Hevekerl et al. 2014a	DSMZ 23081	180 D-glucose	53		3				1		0,1						$8*10^{-3}$	15 mg/L $CuSO_4$ * 7 H_2O,			5 g/L $CaCl_2$ *2 H_2O					1,67 mg/L $FeCl_3$ * 6 H_2O,					400 ml Stirred tank reactor	spores: 1*10^6 spore/ml	N/A	200 ml	N/A	400 rpm	keep 25	3,1	33	7 days		Without $CaCl_2$ fluffy single hyphae, Without CuSO4 changed from pellets to loose hyphae		92,4 g/L IA	
		180 D-glucose	54								0,8																				400 ml Stirred tank reactor	spores: 1*10^6 spore/ml								7 days				86,5 g/L IA	
Hevekerl et al. 2014b	DSMZ 23081	180 D-glucose	53		3				1		0,1						$8*10^{-3}$	15 mg/L $CuSO_4$ * 7 H_2O,			5 g/L $CaCl_2$ *2 H_2O					1,67 mg/L $FeCl_3$ * 6 H_2O,					1,5L Stirred tank reactor	spores: 1*10^6 spore/ml	N/A	1 L	N/A	470 rpm	keep 25	initial:3,1;no control	33	8,8 days				87 g/L IA	fed-batch: D-glucose
		180 D-glucose	59								0,1																				1,5L Stirred tank reactor	spores: 1*10^6 spore/ml		1 L				initial:3,1; set 3,00 after 2 days		12,6 days				146 g/L IA	fed-batch: D-glucose
		180 D-glucose	58								0,8																				1,5L Stirred tank reactor	spores: 1*10^6 spore/ml		1 L				initial:3,1; set 3,00 after 2 days		4,7 days		uniform pellets with 80 μm diameter		129 g/L IA	fed-batch: D-glucose
Karaffa et al. 2015	NRRL 1960	120 D-glucose	65		3				1		0,1						$8*10^{-3}$	15 mg/L $CuSO_4$ * 7 H_2O,			5 g/L $CaCl_2$ *2 H_2O					1,67 mg/L $FeCl_3$ * 6 H_2O,	< 3 ppb				500 ml Erlenmeyer	spores: 5*10^6 spore/ml		100 ml		200		3	33	N/A		small dense pellets		78 g/L IA*	Use ion exchange to remove Mn if any, Mn(II) higher than 5 ppb decr. IA yield
		180 D-glucose	61																								2 pbb				500 ml Erlenmeyer			100 ml								small dense pellets		110 g/L IA	
		180 D-glucose	56																								5 pbb				500 ml Erlenmeyer			100 ml								small dense pellets		100 g/L IA	
		180 D-glucose	50																								10 ppb				500 ml Erlenmeyer			100 ml								long hyphae		90 g/L IA	
		180 D-glucose	33																								250 ppb				500 ml Erlenmeyer			100 ml								long hyphae		60 g/L IA	
		180 D-glucose	25																								2500 pbb				500 ml Erlenmeyer			100 ml								long hyphae		45 g/L IA	
Saha et al. 2017a	99 different strains, best: NRRL 1962	80 D-glucose	61		3				1		0,8						$8*10^{-3}$	15 mg/L $CuSO_4$ * 7 H_2O,			5 g/L $CaCl_2$ *2 H_2O					1,67 mg/L $FeCl_3$ * 6 H_2O,	Limited				125 ml Erlenmeyer	spores: 1*10^6 spore/ml		25 ml		200		3,1	33	7 days				46,6 g/L IA	Use ion exchange to remove Mn if any
		80 D-xylose	48																												125 ml Erlenmeyer			25 ml						7 days				33,6 g/L IA	
		80 L-arabinose	40																												125 ml Erlenmeyer			25 ml						7 days				29,70 g/L IA	
		80 mixed Glu-Xyl-Ara (1:1:1)	45																												125 ml Erlenmeyer			25 ml						10 days				32,90 g/L IA	
Saha et al. 2017b	various, best: NRRL 1971	80 D-galactose	0		3				1		0,8						$8*10^{-3}$	15 mg/L $CuSO_4$ * 7 H_2O,			5 g/L $CaCl_2$ *2 H_2O					1,67 mg/L $FeCl_3$ * 6 H_2O,	Limited				125 ml Erlenmeyer	spores: 1*10^6 spore/ml		25 ml		200		3,1	33	7 days				0 g/L IA	Use ion exchange to remove Mn if any
		80 D-glucose	54																												125 ml Erlenmeyer			25 ml						7 days		pellet, diameter: 46-206 μm		42,6 g/L IA	
		80 D-mannose	47																												125 ml Erlenmeyer			25 ml						9 days		pellet, diameter: 172-432 μm		36,4 g/L IA	
		80 D-xylose	39																												125 ml Erlenmeyer			25 ml						9 days		pellet, diameter: 69-265 μm		30,5 g/L IA	
		80 L-arabinose	33																												125 ml Erlenmeyer			25 ml						9 days		pellet, diameter: 51-204 μm		25,8 g/L IA	
		80 mixed Glu-Man-Xyl-Ara (1:1:1:1)	44																												125 ml Erlenmeyer			25 ml						9 days				35,2 g/L IA	
Krull et al. 2017a	DSMZ 23081	180 D-glucose	46		3				1		0,8						$8*10^{-3}$	15 mg/L $CuSO_4$ * 7 H_2O,			5 g/L $CaCl_2$ *2 H_2O					1,67 mg/L $FeCl_3$ * 6 H_2O,	2,5 ppb				1,5 L Stirred tank reactor	submerged spores: 1*10^6 spore/ml	magnetic stirrer	1 L	N/A	450 rpm	keep 25	initial:3,1; set 3,40 after 2 days	35	7 days		frayed pellets with 80 μm diameter		160 g/L IA	fed-batch: D-glucose, Use ion exchange to remove Mn if any. With 0,8 g/L KH2PO4 was imposibble to perform succesful cultivation with 15L reactor (pellet diameter rose to 200 μm)
		180 D-glucose	57								0,8																2,5 ppb				1,5 L Stirred tank reactor			1 L				initial:3,1; set 3,00 after 2 days		7 days				129 g/L IA	
		180 D-glucose	58								0,1																2,5 ppb				1,5 L Stirred tank reactor			1 L				initial:3,1; set 3,40 after 2 days		12 days				160 g/L IA	
		180 D-glucose	63								0,1																2,5 ppb				1,5 L Stirred tank reactor			1 L				initial:3,1; set 3,00 after 2 days		12 days				144 g/L IA	
		180 D-glucose	56								0,1																2,5 ppb				15 L Stirred tank reactor	submerged spores: 1*10^6 spore/ml	Rushton	10 L	N/A	350	keep 25	initial:3,1; set 3,00 after 2 days		10 days				150 g/L IA	
Krull et al. 2017b	DSMZ 23081	100 sugars (36,6 Glu, 33,1 Xyl, 24,8 Ara, 7,9 Gal, 2,8 Rham)	59		3				1		0,1						$8*10^{-3}$	15 mg/L $CuSO_4$ * 7 H_2O,			5 g/L $CaCl_2$ *2 H_2O					1,67 mg/L $FeCl_3$ * 6 H_2O,	Limited				250 ml shake flasks with buffles	submerged spores: 1*10^6 spore/ml		100 ml		120 rpm		3,1		10 days		fluffy pellets		51,5 g/L IA	Use ion exchange to remove Mn if any
		87,6 Wheat chaff hydrolysate	27																												250 ml shake flasks with buffles			100 ml						6 days		sporulation		23,3 g/L IA	Wheat chaff hydrolysate has not treated
		85,3 detoxicated wheat chaff hydrolysate	41																												250 ml shake flasks with buffles			100 ml						6 days		fluffy pellets		27,7 g/L IA	Wheat chaff hydrolysate has treated with cation exchange
Shin et al. 2017	AN37 mutant	132 sucrose	67*	3				0,5		0,5						$0,2*10^{-3}$	$8,8*10^{-3}$	$0,4*10^{-3}$									0,1 mg/L $MnCl_2$ * 4 H_2O,			2 g/L Yeast extract, 0,005 mg/L Na2MoO4,	5L Stirred tank reactor	5% (v/v) inoculation from mycelia	2 turbine impellers with 6 blades/each 85 mm diameter	3,5 L	1,5 vvm	250 rpm	keep 20	2,1	37	N/A				65 g/L IA*	Overexpress reg, mtt, cad, mfs genes, cad and mfs oe. had positive effect
Wu et al. 2017	CICC40205 mutant	100 wheat bran hydrolysate (eq. To 60 g/L Xyl and 40 g/L Glu)	50*		3			3		0,2						0,16 g/L $FeSO_4$	0,15 g/L $ZnSO_4$													2 g/L CSL	7L Stirred tank reactor	10% (v/v) inoculation from mycelia	4 blade-turbine with 64 mm diameter	5 L	0,06 vvm	400 rpm		2,5	32	120 hours				49,65 g/L IA	
Pedroso et al. 2017	ATCC 10020	Treated hydrolysed rice husk (3,9 g/L Glu; 33, g/L Xyl; 2,1 g/L Ara)	5																											10 g/L yeast extract	125 ml Erlenmeyer	10% (v/v) inoculation from mycelia		20-25ml		100 rpm		6	30	120 hours				2 g/L IA	
Saha et al. 2018b	NRRL 1972	80 sugars (40 g/L Glu; 30 g/L Xyl; 10 g/L Ara)	53		3				1		0,8						$8*10^{-3}$	15 mg/L $CuSO_4$ * 7 H_2O,			5 g/L $CaCl_2$ *2 H_2O					1,67 mg/L $FeCl_3$ * 6 H_2O,	Limited				125 ml Erlenmeyer	spores: 1*10^6 spore/ml		25 ml		200 rpm		3,1	33	7 days				41 g/L IA	Effect of inhibitors to IA production: 0,8 g/l acetic acid 100% inhibition<br>0,8 g/l furfural inhibited fungal growth<br>0,4 g/l HMF 61% inhibition<br>50 ppb Mn 97% inhibition<br>2,4 g/l CaSO4 58% inhibiton
Kreyenschulte et al. 2018	DSMZ 23081	250 D-glucose	42		3				1		0,8						$8*10^{-3}$	15 mg/L $CuSO_4$ * 7 H_2O,			5 g/L $CaCl_2$ *2 H_2O					1,67 mg/L $FeCl_3$ * 6 H_2O,	+1 ppb Mn(II) has added				250 ml Erlenmeyer	spores: 5*10^6 spore/ml		20 ml		300 rpm		3,1	33	192 hours				105 g/L IA	in situ extraction with tryoctilamin isopropyl myristate during the fermentation
Molnár et al. 2018	NRRL 1960	120 D-glucose	38		3				1		0,1						$8*10^{-3}$	15 mg/L $CuSO_4$ * 7 H_2O,			5 g/L $CaCl_2$ *2 H_2O					1,67 mg/L $FeCl_3$ * 6 H_2O,	Limited				9 L Stirred tank reactor	spores: 5*10^6 spore/ml	2 six bladed rushton	6 L	0,75 vvm	N/A	2	3	33	12 days		pellets with same diameter, independent from DO level or rpm		45,9 g/L IA	High oxygen tension requires to express alternative oxidase *aoxA* gene. Alternative respiration correlates with IA overproduction
		120 D-glucose	39																												9 L Stirred tank reactor			6 L		N/A	5			12 days				46,8 g/L IA	
		120 D-glucose	43																												9 L Stirred tank reactor			6 L		N/A	10			12 days				51,10 g/L IA	
		120 D-glucose	54																												9 L Stirred tank reactor			6 L		N/A	20			12 days				65 g/L IA	
		120 D-glucose	61																												9 L Stirred tank reactor			6 L		N/A	30			12 days				73,6 g/L IA	
		120 D-glucose	61																												9 L Stirred tank reactor			6 L		N/A	40			12 days				73,6 g/L IA	

Supplementary Table S3: BLASTp analysis of the proteins encoded by the four genes of the itaconate biosynthesis cluster of *A. terreus* (CadA, MfsA, MttA and the Zn_2Cys_6 transcription factor)

Zn2Cys6 transcription factor

	Description	Max score	Total score	Query cover	E value	Ident	Accession
Select seq XP_001209271.1	predicted protein [Aspergillus terreus NIH2624]	1432	1432	0,99	0.0	1	gi|115385449|XP_001209271.1
Select seq AAD34561.1	regulatory protein [Aspergillus terreus]	1329	1329	0,99	0.0	0,94	gi|4959954|AAD34561.1
Select seq AGV15468.1	C6 transcription factor [Aspergillus terreus]	1272	1272	0,99	0.0	0,91	gi|540850053|AGV15468.1
Select seq OJJ67550.1	hypothetical protein ASPBRDRAFT_47611 [Aspergillus brasiliensis CBS 101740]	406	406	0,82	1E-127	0,38	gi|1111901461|OJJ67550.1
Select seq XP_025564671.1	C6 transcription factor [Aspergillus vadensis CBS 113365]	399	399	0,82	5E-125	0,37	gi|1422897819|XP_025564671.1
Select seq XP_025383818.1	C6 transcription factor [Aspergillus eucalypticola CBS 122712]	399	399	0,82	7E-125	0,38	gi|1418983954|XP_025383818.1
Select seq OJI91586.1	hypothetical protein ASPTUDRAFT_53222 [Aspergillus tubingensis CBS 134.48]	399	399	0,82	1E-124	0,37	gi|1111704661|OJI91586.1
Select seq OJZ81380.1	hypothetical protein ASPFODRAFT_199484 [Aspergillus luchuensis CBS 106.47]	397	397	0,82	3E-124	0,37	gi|1114056370|OJZ81380.1
Select seq GAQ45093.1	C6 transcription factor [Aspergillus niger]	397	397	0,82	3E-124	0,37	gi|966762176|GAQ45093.1
Select seq GAA88110.1	C6 transcription factor [Aspergillus kawachii IFO 4308]	397	397	0,82	4E-124	0,37	gi|358371502|GAA88110.1
Select seq XP_025509919.1	C6 transcription factor [Aspergillus piperis CBS 112811]	397	397	0,82	4E-124	0,37	gi|1419169134|XP_025509919.1
Select seq XP_025484389.1	C6 transcription factor [Aspergillus neoniger CBS 115656]	396	396	0,82	8E-124	0,37	gi|1419045609|XP_025484389.1
Select seq XP_025540520.1	C6 transcription factor [Aspergillus costaricaensis CBS 115574]	395	395	0,82	2E-123	0,37	gi|1422749100|XP_025540520.1
Select seq SPB52049.1	unnamed protein product [Aspergillus niger]	393	393	0,82	1E-122	0,38	gi|1335395452|SPB52049.1
Select seq XP_025454603.1	hypothetical protein BO96DRAFT_474667 [Aspergillus lacticoffeatus CBS 101883]	393	393	0,82	1E-122	0,38	gi|1419029089|XP_025454603.1
Select seq RDK37711.1	hypothetical protein M752DRAFT_258392 [Aspergillus phoenicis ATCC 13157]	392	392	0,82	2E-122	0,38	gi|1440836602|RDK37711.1
Select seq RDH30871.1	fungal-specific transcription factor domain-domain-containing protein [Aspergillus welwitschiae]	392	392	0,82	2E-122	0,38	gi|1435863580|RDH30871.1
Select seq PYH87539.1	hypothetical protein BO71DRAFT_404581 [Aspergillus ellipticus CBS 707.79]	389	389	0,82	2E-121	0,37	gi|1399676227|PYH87539.1
Select seq XP_025572864.1	hypothetical protein BO80DRAFT_412453 [Aspergillus ibericus CBS 121593]	388	388	0,79	9E-121	0,39	gi|1422686996|XP_025572864.1
Select seq EHA18675.1	hypothetical protein ASPNIDRAFT_120084 [Aspergillus niger ATCC 1015]	382	382	0,78	2E-119	0,38	gi|350630302|EHA18675.1
Select seq PYI04679.1	hypothetical protein BO78DRAFT_372660 [Aspergillus sclerotiicarbonarius CBS 121057]	382	382	0,79	1E-118	0,38	gi|1399693915|PYI04679.1
Select seq GAT22624.1	C6 transcription factor [Aspergillus luchuensis]	379	379	0,82	2E-117	0,36	gi|1002330277|GAT22624.1
Select seq XP_025463847.1	hypothetical protein BO94DRAFT_538547 [Aspergillus sclerotioniger CBS 115572]	378	378	0,79	2E-117	0,37	gi|1423545524|XP_025463847.1
Select seq XP_025466979.1	hypothetical protein BO94DRAFT_624585 [Aspergillus sclerotioniger CBS 115572]	378	378	0,82	5E-117	0,36	gi|1423531666|XP_025466979.1
Select seq OOF94390.1	hypothetical protein ASPCADRAFT_23956 [Aspergillus carbonarius ITEM 5010]	371	371	0,79	1E-115	0,38	gi|1147628823|OOF94390.1
Select seq OOF90250.1	hypothetical protein ASPCADRAFT_179433 [Aspergillus carbonarius ITEM 5010]	374	374	0,79	2E-115	0,37	gi|1147624630|OOF90250.1
Select seq PYI12190.1	hypothetical protein BO78DRAFT_425429 [Aspergillus sclerotiicarbonarius CBS 121057]	373	373	0,83	3E-115	0,37	gi|1399701480|PYI12190.1
Select seq OWW29015.1	Fungal specific transcription factor domain family protein [Aspergillus niger]	369	369	0,72	7E-114	0,4	gi|1213319780|OWW29015.1
Select seq XP_001274697.1	C6 transcription factor, putative [Aspergillus clavatus NRRL 1]	367	367	0,87	2E-112	0,36	gi|121714172|XP_001274697.1
Select seq XP_024686342.1	putative C6 transcription factor [Aspergillus novofumigatus IBT 16806]	363	363	0,83	1E-110	0,36	gi|1383809747|XP_024686342.1
Select seq OXN02020.1	hypothetical protein CDV58_09024 [Aspergillus fumigatus]	360	360	0,81	1E-109	0,37	gi|1220541256|OXN02020.1
Select seq KMK62905.1	C6 transcription factor [Aspergillus fumigatus Z5]	360	360	0,81	1E-109	0,37	gi|846917135|KMK62905.1
Select seq KEY79761.1	transcription factor C6 [Aspergillus fumigatus var. RP-2014]	358	358	0,81	9E-109	0,36	gi|666432173|KEY79761.1
Select seq GAO83017.1	phosphate-repressible phosphate permease [Aspergillus udagawae]	359	359	0,8	2E-107	0,37	gi|849274644|GAO83017.1
Select seq RHZ55428.1	hypothetical protein CDV56_107678 [Aspergillus thermomutatus]	368	368	0,81	4E-107	0,38	gi|1475561495|RHZ55428.1
Select seq XP_025576304.1	hypothetical protein BO80DRAFT_501488 [Aspergillus ibericus CBS 121593]	350	350	0,79	3E-106	0,36	gi|1422660679|XP_025576304.1
Select seq XP_001259243.1	C6 transcription factor, putative [Aspergillus fischeri NRRL 181]	351	351	0,79	5E-106	0,36	gi|119477081|XP_001259243.1
Select seq EDP52162.1	C6 transcription factor, putative [Aspergillus fumigatus A1163]	350	350	0,81	2E-105	0,36	gi|159127046|EDP52162.1
Select seq XP_753228.1	C6 transcription factor, putative [Aspergillus fumigatus Af293]	350	350	0,81	2E-105	0,36	gi|70996947|XP_753228.1
Select seq GAQ09918.1	phosphate-repressible phosphate permease pho-4 [Aspergillus lentulus]	357	357	0,79	8E-105	0,37	gi|952549475|GAQ09918.1
Select seq XP_001393191.2	hypothetical protein ANI_1_2492074 [Aspergillus niger CBS 513.88]	330	330	0,63	5E-101	0,4	gi|317031324|XP_001393191.2
Select seq XP_025520528.1	hypothetical protein BO85DRAFT_493863 [Aspergillus piperis CBS 112811]	320	320	0,8	1E-95	0,36	gi|1419146302|XP_025520528.1
Select seq XP_025563140.1	hypothetical protein BO88DRAFT_443931 [Aspergillus vadensis CBS 113365]	319	319	0,8	4E-95	0,37	gi|1422905937|XP_025563140.1
Select seq GAT27717.1	C6 transcription factor [Aspergillus luchuensis]	318	318	0,79	4E-95	0,37	gi|1002325240|GAT27717.1
Select seq OCL12672.1	hypothetical protein AOQ84DRAFT_311826 [Glonium stellatum]	321	321	0,79	4E-94	0,32	gi|1046807280|OCL12672.1
Select seq XP_025388786.1	hypothetical protein BO83DRAFT_443670 [Aspergillus eucalypticola CBS 122712]	309	309	0,82	2E-91	0,36	gi|1418963808|XP_025388786.1
Select seq XP_025482403.1	hypothetical protein BO87DRAFT_451550 [Aspergillus neoniger CBS 115656]	307	307	0,82	8E-91	0,35	gi|1419048203|XP_025482403.1
Select seq RDK37969.1	hypothetical protein M752DRAFT_286674 [Aspergillus phoenicis ATCC 13157]	307	307	0,82	2E-90	0,34	gi|1440836862|RDK37969.1
Select seq OJI88269.1	hypothetical protein ASPTUDRAFT_80736 [Aspergillus tubingensis CBS 134.48]	306	306	0,79	4E-90	0,36	gi|1111701340|OJI88269.1
Select seq XP_025458838.1	hypothetical protein BO96DRAFT_430694 [Aspergillus lacticoffeatus CBS 101883]	305	305	0,82	6E-90	0,34	gi|1419021717|XP_025458838.1
Select seq XP_001393933.1	C6 transcription factor [Aspergillus niger CBS 513.88]	305	305	0,82	6E-90	0,34	gi|145242720|XP_001393933.1
Select seq RDH32646.1	fungal-specific transcription factor domain-domain-containing protein [Aspergillus welwitschiae]	305	305	0,82	1E-89	0,34	gi|1435865513|RDH32646.1
Select seq XP_025545111.1	hypothetical protein BO79DRAFT_134724 [Aspergillus costaricaensis CBS 115574]	299	299	0,79	1E-87	0,35	gi|1422711295|XP_025545111.1
Select seq PYH96173.1	hypothetical protein BO71DRAFT_428393 [Aspergillus ellipticus CBS 707.79]	270	270	0,82	4E-77	0,3	gi|1399685246|PYH96173.1
Select seq OJZ82168.1	hypothetical protein ASPFODRAFT_143720 [Aspergillus luchuensis CBS 106.47]	265	265	0,58	1E-76	0,39	gi|1114057161|OJZ82168.1
Select seq GAA88206.1	C6 transcription factor [Aspergillus kawachii IFO 4308]	261	261	0,58	2E-75	0,38	gi|358371599|GAA88206.1
Select seq EHA28564.1	hypothetical protein ASPNIDRAFT_43132 [Aspergillus niger ATCC 1015]	262	262	0,76	4E-74	0,33	gi|350640211|EHA28564.1
Select seq SPB43169.1	unnamed protein product [Aspergillus niger]	262	262	0,76	7E-74	0,33	gi|1335405555|SPB43169.1
Select seq OJJ69336.1	hypothetical protein ASPBRDRAFT_657139 [Aspergillus brasiliensis CBS 101740]	257	257	0,66	2E-72	0,34	gi|1111903353|OJJ69336.1
Select seq OWW33057.1	Fungal specific transcription factor domain family protein [Aspergillus niger]	262	262	0,76	2E-71	0,33	gi|1213323834|OWW33057.1
Select seq GAT27716.1	C6 transcription factor [Aspergillus luchuensis]	204	204	0,58	1E-54	0,34	gi|1002325239|GAT27716.1
Select seq PYH96176.1	hypothetical protein BO71DRAFT_397418 [Aspergillus ellipticus CBS 707.79]	158	158	0,79	1E-36	0,25	gi|1399685249|PYH96176.1
Select seq PGH16701.1	hypothetical protein AJ79_01574 [Helicocarpus griseus UAMH5409]	150	150	0,82	6E-34	0,23	gi|1264178586|PGH16701.1
Select seq SCO84911.1	related to GAL4-like transcriptional activator [Fusarium oxysporum]	147	147	0,63	6E-33	0,25	gi|1252235297|SCO84911.1
Select seq EXL77005.1	hypothetical protein FOPG_08314 [Fusarium oxysporum f. sp. conglutinans race 2 54008]	147	147	0,63	8E-33	0,25	gi|591444477|EXL77005.1
Select seq EGU85665.1	hypothetical protein FOXB_03811 [Fusarium oxysporum Fo5176]	147	147	0,63	8E-33	0,25	gi|342885683|EGU85665.1
Select seq RAO67949.1	hypothetical protein BHQ10_003961 [Talaromyces amestolkiae]	146	146	0,75	9E-33	0,24	gi|1408931014|RAO67949.1
Select seq EXA48228.1	hypothetical protein FOVG_05053 [Fusarium oxysporum f. sp. pisi HDV247]	146	146	0,63	1E-32	0,25	gi|587750512|EXA48228.1
Select seq EXK92676.1	hypothetical protein FOQG_05738 [Fusarium oxysporum f. sp. raphani 54005]	146	146	0,63	1E-32	0,25	gi|590065152|EXK92676.1
Select seq EXA48227.1	hypothetical protein FOVG_05053 [Fusarium oxysporum f. sp. pisi HDV247]	146	146	0,63	1E-32	0,25	gi|587750511|EXA48227.1
Select seq EXK92675.1	hypothetical protein FOQG_05738 [Fusarium oxysporum f. sp. raphani 54005]	146	146	0,63	1E-32	0,25	gi|590065151|EXK92675.1
Select seq EXA01396.1	hypothetical protein FOWG_01272 [Fusarium oxysporum f. sp. lycopersici MN25]	146	146	0,63	1E-32	0,25	gi|587730058|EXA01396.1
Select seq XP_018245257.1	hypothetical protein FOXG_19815 [Fusarium oxysporum f. sp. lycopersici 4287]						

Mitochondrial cis-aconitate transporter

	Description	Max score	Total score	Query cover	E value	Ident	Accession
Select seq XP_001209272.1	predicted protein [Aspergillus terreus NIH2624]	594	594	0,99	0.0	1	gi|115385451|XP_001209272.1
Select seq AAD34562.1	unknown [Aspergillus terreus]	588	588	0,99	0.0	0,98	gi|4959955|AAD34562.1
Select seq CDG56263.1	Mitochondrial transporter [Aspergillus terreus]	586	586	0,99	0.0	0,98	gi|548954875|CDG56263.1
Select seq GAP93451.1	putative tricarboxylate transport protein [Rosellinia necatrix]	244	244	0,93	2E-76	0,46	gi|949383493|GAP93451.1
Select seq ESZ96799.1	tricarboxylate transport protein [Sclerotinia borealis F-4128]	243	243	0,93	2E-75	0,45	gi|563296602|ESZ96799.1
Select seq KKF97159.1	putative mitochondrial carrier C19G12.05 [Ceratocystis platani]	237	237	0,97	2E-73	0,42	gi|814602616|KKF97159.1
Select seq ORY67098.1	putative tricarboxylate transporter mitochondrial carrier protein [Pseudomassariella vexata]	237	237	0,89	2E-73	0,45	gi|1183442933|ORY67098.1
Select seq KIW69373.1	hypothetical protein PV04_05254 [Phialophora americana]	236	236	0,9	4E-73	0,45	gi|759292957|KIW69373.1
Select seq KFA70073.1	hypothetical protein S40285_03300 [Stachybotrys chlorohalonata IBT 40285]	236	236	0,89	6E-73	0,46	gi|667729241|KFA70073.1
Select seq GAM43162.1	hypothetical protein TCE0_047f17744 [Talaromyces cellulolyticus]	233	233	0,71	9E-73	0,56	gi|748550924|GAM43162.1
Select seq PMD14049.1	putative tricarboxylate transporter mitochondrial carrier protein [Pezoloma ericae]	235	235	0,89	1E-72	0,45	gi|1325530666|PMD14049.1
Select seq OTA65785.1	putative tricarboxylate transporter mitochondrial carrier protein [Hypoxylon sp. EC38]	234	234	0,89	1E-72	0,46	gi|1190873816|OTA65785.1
Select seq XP_956064.3	mitochondrial tricarboxylate transporter [Neurospora crassa OR74A]	235	235	0,89	1E-72	0,46	gi|758998337|XP_956064.3
Select seq GAW23758.1	hypothetical protein ANO14919_133340 [fungal sp. No.14919]	234	234	0,93	1E-72	0,45	gi|1141554307|GAW23758.1
Select seq KEY65502.1	hypothetical protein S7711_09292 [Stachybotrys chartarum IBT 7711]	234	234	0,89	2E-72	0,46	gi|666399674|KEY65502.1
Select seq XP_007294727.1	hypothetical protein MBM_06838 [Marssonina brunnea f. sp. 'multigermtubi' MB_m1]	234	234	0,92	2E-72	0,45	gi|597583341|XP_007294727.1

cis-aconitate permease

	Description	Max score	Total score	Query cover	E value	Ident	Accession
Select seq XP_001209274.1	predicted protein [Aspergillus terreus NIH2624]	781	781	0,99	0.0	1	gi|115385455|XP_001209274.1
Select seq AGV15467.1	major facilitator superfamily transporter [Aspergillus terreus]	709	709	0,95	0.0	0,95	gi|540850040|AGV15467.1
Select seq AAD34564.1	unknown [Aspergillus terreus]	706	706	0,95	0.0	0,92	gi|4959957|AAD34564.1
Select seq CDG56264.1	plasmalemma transporter [Aspergillus terreus]	704	704	0,95	0.0	0,92	gi|548954877|CDG56264.1
Select seq XP_002382138.1	MFS transporter, putative [Aspergillus flavus NRRL3357]	542	542	0,95	0.0	0,71	gi|238501808|XP_002382138.1
Select seq XP_001819137.1	unnamed protein product [Aspergillus oryzae RIB40]	542	542	0,95	0.0	0,71	gi|169769334|XP_001819137.1
Select seq KOC10872.1	MFS transporter [Aspergillus flavus AF70]	542	542	0,95	0.0	0,71	gi|914756707|KOC10872.1
Select seq KJK63559.1	Major Facilitator Superfamily protein [Aspergillus parasiticus SU-1]	539	539	0,95	0.0	0,71	gi|770305745|KJK63559.1
Select seq PIG79507.1	MFS transporter [Aspergillus arachidicola]	539	539	0,95	0.0	0,7	gi|1274066857|PIG79507.1
Select seq OJJ30637.1	hypothetical protein ASPWEDRAFT_121132 [Aspergillus wentii DTO 134E9]	502	502	0,9	3E-174	0,7	gi|1111847758|OJJ30637.1
Select seq XP_025570899.1	synaptic vesicle transporter [Aspergillus ibericus CBS 121593]	502	502	0,93	2E-173	0,66	gi|1422700925|XP_025570899.1
Select seq XP_024705675.1	putative MFS transporter [Aspergillus steynii IBT 23096]	501	501	0,94	4E-173	0,67	gi|1383867139|XP_024705675.1
Select seq XP_022389980.1	MFS transporter [Aspergillus bombycis]	497	497	0,95	8E-172	0,67	gi|1245870252|XP_022389980.1
Select seq SPB51793.1	unnamed protein product [Aspergillus niger]	496	496	0,95	9E-171	0,65	gi|1335397703|SPB51793.1
Select seq RDH31313.1	major facilitator superfamily domain-containing protein [Aspergillus welwitschiae]	495	495	0,95	2E-170	0,65	gi|1435864067|RDH31313.1
Select seq XP_025465081.1	synaptic vesicle transporter [Aspergillus sclerotioniger CBS 115572]	494	494	0,92	3E-170	0,66	gi|1423540131|XP_025465081.1
Select seq PYI01862.1	synaptic vesicle transporter [Aspergillus sclerotiicarbonarius CBS 121057]	494	494	0,94	4E-170	0,65	gi|1399691057|PYI01862.1
Select seq GAT28158.1	MFS transporter [Aspergillus luchuensis]	490	490	0,96	2E-169	0,64	gi|1002324965|GAT28158.1
Select seq OOF98646.1	hypothetical protein ASPCADRAFT_394061 [Aspergillus carbonarius ITEM 5010]	492	492	0,93	3E-169	0,66	gi|1147633086|OOF98646.1
Select seq OWW38117.1	major facilitator superfamily protein [Aspergillus niger]	484	484	0,95	4E-167	0,65	gi|1213328913|OWW38117.1
Select seq OJI83271.1	hypothetical protein ASPTUDRAFT_44601 [Aspergillus tubingensis CBS 134.48]	484	484	0,95	2E-166	0,65	gi|1111696318|OJI83271.1
Select seq XP_025435236.1	putative MFS transporter [Aspergillus saccharolyticus JOP 1030-1]	484	484	0,89	6E-166	0,68	gi|1418997297|XP_025435236.1
Select seq RJE23400.1	Mfs transporter [Aspergillus sclerotialis]	480	480	0,94	6E-165	0,65	gi|1480717177|RJE23400.1
Select seq CDM36253.1	Major facilitator superfamily [Penicillium roqueforti FM164]	479	479	0,94	1E-164	0,65	gi|584407429|CDM36253.1
Select seq PYI17181.1	putative MFS transporter [Aspergillus violaceofuscus CBS 115571]	476	476	0,89	1E-163	0,67	gi|1399706627|PYI17181.1
Select seq XP_025525791.1	putative MFS transporter [Aspergillus japonicus CBS 114.51]	476	476	0,89	2E-163	0,67	gi|1422768934|XP_025525791.1
Select seq XP_001260160.1	MFS transporter, putative [Aspergillus fischeri NRRL 181]	475	475	0,9	3E-163	0,66	gi|119480263|XP_001260160.1
Select seq RHZ44295.1	hypothetical protein CDV56_103516 [Aspergillus thermomutatus]	472	472	0,94	6E-163	0,64	gi|1475549220|RHZ44295.1
Select seq XP_025486417.1	synaptic vesicle transporter [Aspergillus uvarum CBS 121591]	474	474	0,89	7E-163	0,66	gi|1419143803|XP_025486417.1
Select seq XP_025498122.1	synaptic vesicle transporter [Aspergillus aculeatinus CBS 121060]	477	477	0,9	7E-163	0,66	gi|1419192853|XP_025498122.1
Select seq RAK73607.1	MFS general substrate transporter [Aspergillus fijiensis CBS 313.89]	476	476	0,9	9E-163	0,65	gi|1407778741|RAK73607.1
Select seq KZN85476.1	putative transporter [Penicillium chrysogenum]	474	474	0,94	2E-162	0,65	gi|1021538118|KZN85476.1
Select seq XP_025440350.1	synaptic vesicle transporter [Aspergillus brunneoviolaceus CBS 621.78]	476	476	0,9	2E-162	0,66	gi|1419085577|XP_025440350.1
Select seq OQD65144.1	hypothetical protein PENPOL_c006G05830 [Penicillium polonicum]	473	473	0,94	6E-162	0,64	gi|1168062891|OQD65144.1
Select seq KUM57941.1	hypothetical protein ACN42_g9230 [Penicillium freii]	473	473	0,94	7E-162	0,64	gi|973783807|KUM57941.1
Select seq XP_002564939.1	Pc22g09270 [Penicillium rubens Wisconsin 54-1255]	471	471	0,88	2E-161	0,69	gi|255948344|XP_002564939.1
Select seq GAO90936.1	uncharacterized transporter C750.02c/PB2B2.16c [Aspergillus udagawae]	470	470	0,93	4E-161	0,64	gi|849266599|GAO90936.1

cis-aconitate decarboxylase

	Description	Max score	Total score	Query cover	E value	Ident	Accession
Select seq XP_001209273.1	predicted protein [Aspergillus terreus NIH2624]	918	918	1	0.0	1	gi|115385453|XP_001209273.1
Select seq ALI59896.1	cis-aconitate decarboxylase [synthetic construct]	916	916	0,99	0.0	1	gi|937561455|ALI59896.1
Select seq AAD34563.1	unknown [Aspergillus terreus]	906	906	1	0.0	0,99	gi|4959956|AAD34563.1
Select seq AJE61287.1	cis-aconitate decarboxylase [synthetic construct]	904	904	1	0.0	0,98	gi|748758663|AJE61287.1
Select seq B3IUN8.1	RecName: Full=Cis-aconitate decarboxylase; Short=CAD; AltName: Full=Aconitate decarboxylase; AltNa	898	898	1	0.0	0,98	gi|527525149|B3IUN8.1
Select seq AWM95822.1	cis-aconitate decarboxylase [synthetic construct]	896	896	0,99	0.0	0,98	gi|1391784636|AWM95822.1
Select seq ASR91696.1	cis-aconitic acid decarboxylase [Aspergillus terreus]	860	860	0,93	0.0	0,99	gi|1228198436|ASR91696.1
Select seq ASR91697.1	cis-aconitic acid decarboxylase [Aspergillus terreus]	843	843	0,93	0.0	0,99	gi|1228198438|ASR91697.1
Select seq PCH01471.1	MmgE/PrpD [Penicillium occitanis]	547	547	0,97	0.0	0,6	gi|1247112923|PCH01471.1
Select seq GAM43161.1	immune-responsive protein [Talaromyces cellulolyticus]	471	471	0,86	1E-160	0,6	gi|748550923|GAM43161.1
Select seq RAO68128.1	hypothetical protein BHQ10_004140 [Talaromyces amestolkiae]	470	470	0,86	2E-160	0,61	gi|1408931193|RAO68128.1
Select seq XP_022385936.1	immune-responsive protein [Aspergillus bombycis]	471	471	0,97	7E-160	0,55	gi|1245886412|XP_022385936.1
Select seq XP_015412298.1	immune-responsive protein [Aspergillus nomius NRRL 13137]	470	470	0,97	1E-159	0,55	gi|992231797|XP_015412298.1
Select seq GAW23112.1	hypothetical protein ANO14919_126620 [fungal sp. No.14919]	471	471	0,99	3E-159	0,53	gi|1141555027|GAW23112.1
Select seq KOC13246.1	immune-responsive protein [Aspergillus flavus AF70]	468	468	0,97	1E-158	0,55	gi|914759400|KOC13246.1
Select seq XP_001827196.1	unnamed protein product [Aspergillus oryzae RIB40]	468	468	0,97	2E-158	0,55	gi|169785471|XP_001827196.1
Select seq OOO07454.1	MmgE/PrpD family protein [Aspergillus oryzae]	466	466	0,97	5E-158	0,55	gi|1150472687|OOO07454.1
Select seq RAQ45391.1	immune-responsive protein [Aspergillus flavus]	466	466	0,97	1E-157	0,54	gi|1409521840|RAQ45391.1
Select seq PIG84203.1	immune-responsive protein [Aspergillus arachidicola]	465	465	0,96	1E-157	0,55	gi|1274072286|PIG84203.1
Select seq GAP92989.1	putative immune-responsive protein [Rosellinia necatrix]	462	462	0,97	3E-156	0,54	gi|949383977|GAP92989.1
Select seq XP_020051550.1	hypothetical protein ASPACDRAFT_36774 [Aspergillus aculeatus ATCC 16872]						

Supplementary Table S4: Distribution of genes putatively involved in citric acid biosynthesis within the *A. niger* genome.
Gene order on scaffolds and their putative function was retrieved from https://genome.jgi.doe.gov/Aspni7/Aspni7.home.html.
Colour highlights gene couples in close (sometimes immediate) vicinity

scaffold	begin	end	orientation	protein ID	putative function
chr_101	618762	618933	+	1155979	Dihydrolipoamide transacylase (alpha-keto acid dehydrogenase E2 subunit)
chr_101	850042	850130	+	126525	Citrate synthase
chr_101	914412	914475	+	1181034	Aconitase/homoaconitase (aconitase superfamily)
chr_101	1566278	1566520	-	1141644	Mitochondrial tricarboxylate/dicarboxylate carrier proteins
chr_101	1569453	1569859	-	1141647	Citrate synthase
chr_102	86146	86330	+	1141712	Succinyl-CoA synthetase, beta subunit
chr_102	474994	475461	-	1115775	Mitochondrial carrier protein - Rim2p/Mrs12p
chr_102	1087408	1088036	+	1202809	Succinate dehydrogenase, Fe-S protein subunit
chr_102	1089406	1090272	-	211172	Fructose 1,6-bisphosphate aldolase
chr_102	1198764	1198885	+	1141976	Triosephosphate isomerase
chr_102	1544361	1544989	-	1080611	Dihydroxyacetone kinase/glycerone kinase
chr_102	1641469	1641672	+	1142076	Mitochondrial oxaloacetate carrier protein
chr_202	49069	49633	-	1005336	Pyruvate dehydrogenase E1, beta subunit
chr_202	70433	70759	+	1116881	Succinyl-CoA:alpha-ketoacid-CoA transferase
chr_201	138697	140346	+	1170740	Hexokinase
chr_202	416199	416884	+	1162391	Predicted phosphoglycerate mutase
chr_202	847360	847667	+	1162479	Succinyl-CoA:alpha-ketoacid-CoA transferase
chr_202	1587357	1587950	+	1117437	Glycerol-3-phosphate dehydrogenase/dihydroxyacetone 3-phosphate reductase
chr_202	2357460	2357960	-	1102128	Isocitrate lyase
chr_202	2507044	2507395	+	1117800	Citrate synthase
chr_202	3490660	3490895	+	1143065	Succinate dehydrogenase membrane anchor subunit and related proteins
chr_301	123860	124130	-	1133038	Fructose 1,6-bisphosphate aldolase
chr_302	633824	634163	+	1163625	Mitochondrial carrier protein CGI-69
chr_302	699929	700467	-	42353	Isocitrate lyase
chr_302	992176	992418	-	1119446	Glyceraldehyde 3-phosphate dehydrogenase
chr_302	1219063	1219275	-	1119507	Isocitrate lyase
chr_302	1269375	1269561	-	1133804	Fumarase
chr_302	1305382	1305587	-	1183468	Phosphoglycerate mutase
chr_302	1421888	1422347	+	1143538	Hexokinase
chr_304	32072	32192	-	1143651	NAD-dependent malate dehydrogenase
chr_304	108197	109230	+	1104257	Aconitase/homoaconitase (aconitase superfamily)
chr_304	538435	538520	+	1143782	Citrate synthase
chr_304	730767	732675	-	1120162	Pyruvate carboxylase
chr_304	768578	769096	+	1164105	Isocitrate lyase
chr_304	1412430	1413992	-	200758	Hexokinase
chr_304	1777662	1777826	-	1157919	Glycerol-3-phosphate dehydrogenase/dihydroxyacetone 3-phosphate reductase
chr_304	1851227	1851907	-	1104964	Isocitrate lyase
chr_401	444551	444596	+	1087302	Fructose-6-phosphate 2-kinase/fructose-2,6-biphosphatase
chr_401	461235	462101	-	1144118	NAD-dependent malate dehydrogenase
chr_401	719159	719214	+	1144183	Succinate dehydrogenase, cytochrome b subunit
chr_401	1207863	1207964	+	1184412	Predicted phosphoglycerate mutase
chr_401	1895485	1895858	+	1164837	Isocitrate lyase
chr_401	2086498	2086566	-	1184650	Pyruvate kinase
chr_401	2225566	2225750	+	1019169	Pyruvate dehydrogenase E1, alpha subunit
chr_401	2301355	2301635	+	1087983	Fructose-6-phosphate 2-kinase/fructose-2,6-biphosphatase
chr_401	2431061	2432196	-	1019508	Mitochondrial carrier protein
chr_402	468949	469889	-	1158342	Mitochondrial oxoglutarate/malate carrier proteins
chr_402	760214	761375	-	1144773	Triosephosphate isomerase
chr_402	1356940	1358508	+	1122095	Fumarate reductase, flavoprotein subunit
chr_402	1882561	1883434	-	1145051	Fructose 1,6-bisphosphate aldolase
chr_402	1917583	1917743	+	1215111	Succinate dehydrogenase, flavoprotein subunit
chr_402	2849832	2850782	-	1175567	Aconitase/homoaconitase (aconitase superfamily)
chr_402	2860135	2860249	+	37369	Mitochondrial tricarboxylate/dicarboxylate carrier proteins
chr_402	2961457	2962238	-	1145291	Aconitase/homoaconitase (aconitase superfamily)
chr_402	3139535	3139597	+	1175666	NADP-dependent isocitrate dehydrogenase
chr_402	3214235	3214276	+	1136340	Succinate dehydrogenase, flavoprotein subunit
chr_402	3610360	3611436	-	1145456	Hexokinase
chr_501	212892	213833	-	1145545	Isocitrate lyase
chr_501	713577	713848	+	1145655	Succinyl-CoA synthetase, alpha subunit
chr_502	361425	362634	-	1176346	Hexokinase
chr_502	415758	415874	+	1145793	Glyceraldehyde 3-phosphate dehydrogenase
chr_502	959592	959728	+	1145895	Phosphoglycerate mutase
chr_502	1531744	1531818	+	1146031	Glucose-6-phosphate isomerase
chr_502	1597392	1599299	-	1218960	Aconitase/homoaconitase (aconitase superfamily)
chr_502	1646615	1646713	+	1124768	Phosphoglycerate mutase
chr_503	131474	131571	-	1176833	Fumarate reductase, flavoprotein subunit
chr_601	1120054	1120398	-	1110181	Mitochondrial carrier protein
chr_602	1199346	1199697	+	1146854	2-oxoglutarate dehydrogenase, E1 subunit
chr_602	1325472	1325681	+	1146884	Fructose-1,6-bisphosphatase
chr_602	573236	573327	+	1031996	Pyruvate carboxylase
chr_603	242727	243340	-	1223085	Mitochondrial tricarboxylate/dicarboxylate carrier proteins
chr_701	186572	187419	-	1111634	ATP-citrate lyase
chr_701	191591	191749	+	1147138	ATP-citrate lyase
chr_701	677134	677182	-	1178527	Mitochondrial oxoglutarate/malate carrier proteins
chr_701	837251	837540	-	1139152	Predicted phosphoglycerate mutase
chr_701	1211103	1211231	+	1112134	Pyruvate dehydrogenase E1, alpha subunit
chr_701	1791847	1792193	-	48047	NAD-dependent malate dehydrogenase
chr_701	2780926	2780958	+	1179216	Mitochondrial tricarboxylate/dicarboxylate carrier proteins
chr_701	2789338	2789513	-	1037649	Dihydrolipoamide succinyltransferase (2-oxoglutarate dehydrogenase, E2 subunit)
chr_801	1468102	1468430	+	1113563	Enolase
chr_801	20139	20174	+	1179236	Mitochondrial tricarboxylate/dicarboxylate carrier proteins
chr_801	329300	330446	+	1128684	Pyrophosphate-dependent phosphofructo-1-kinase
chr_802	32599	33038	+	1098652	Glycerol-3-phosphate dehydrogenase
chr_802	299720	299883	+	1113742	Mitochondrial oxodicarboxylate carrier protein
chr_802	400268	400297	+	177364	Mitochondrial carrier protein PET8
chr_802	507043	507273	+	1148178	3-phosphoglycerate kinase
chr_802	611593	611727	+	1188917	Predicted phosphoglycerate mutase
chr_802	693654	693822	+	1168950	Succinyl-CoA synthetase, alpha subunit
chr_802	1147344	1147617	-	1140669	Fumarate reductase, flavoprotein subunit
chr_802	2534574	2535224	-	1148603	Aconitase/homoaconitase (aconitase superfamily)
chr_802	2540215	2540259	-	1180573	Mitochondrial carrier protein
chr_802	2639456	2639744	-	1148640	Mitochondrial carrier protein MRS3/4
chr_802	2743724	2743786	+	1155720	Succinyl-CoA:alpha-ketoacid-CoA transferase
chr_802	2841277	2843010	-	1130266	Fumarate reductase, flavoprotein subunit
chr_802	2982915	2985243	+	1155769	2-oxoglutarate dehydrogenase, E1 subunit
chr_802	2985955	2986776	-	1155770	Hexokinase
chr_802	3081000	3081314	+	1114699	Citrate synthase
scaffold_10	5001	5816	-	46158	Predicted phosphoglycerate mutase

Supplementary Table S5: Reference literature containing input data used to create **Figure 2** (trilateral relationship between carbon source concentration, aeration and yield of citric acid).

Steel R, Lentz CP, Martin SM (1955) Submerged citric acid fermentation of sugar beet molasses: increase in scale. Can. J. Microbiol. 1:299-311.

Clark DS, Lentz CP (1961) Submerged citric acid fermentation of sugar beet molasses: effect of pressure and recirculation of oxygen. Can. J. Microbiol. 7:447-453.

DS Clark, K Ito, H Horitsu (1966) Effect of manganese and other heavy metals on submerged citric acid fermentation of molasses. Biotechnol. Bioeng. 8:465-471.

Zhang A, Röhr M (2002) Citric acid fermentation and heavy metal ions II. The action of elevated manganese ion concentrations. Acta Biotechnol. 22:375-382.

Papagianni M, Wayman F, Mattey M (2005) Fate and role of ammonium ions during fermentation of citric acid by *Aspergillus niger*. Appl. Environ. Microbiol. 71:7178-7186.

Papagianni M, Mattey M (2006) Morphological development of *Aspergillus niger* in submerged citric acid fermentation as a function of the spore inoculum level. Application of neural network and cluster analysis for characterization of mycelial morphology. Microb. Cell Fact. 5:3.

Papagianni M (2006) Quantification of the fractal nature of mycelial aggregation in *Aspergillus niger* submerged cultures. Microb. Cell Fact. 5:5.

Supplementary Table S6: References containing input data used for **Figure 3** (trilateral relationship between carbon source concentration, aeration and yield of itaconic acid).

Pfeifer VF, Vojnovich C, Heger EN (1952) Itaconic acid by fermentation with *Aspergillus terreus*. Ind. Eng. Chem. 44:2975–2980

Kautola H, Vahvaselkä M, Linko YY, Linko P (1985) Itaconic acid production by immobilized *Aspergillus terreus* from xylose and glucose. Biotechnol. Lett. 7:167-172.

Kautola H, Vassilev N, Linko YY (1990) Continuous itaconic acid production by immobilized biocatalysts. J. Biotechnol. 13:315-323.

Vassilev N, Kautola H, Linko YY (1992) Immobilized *Aspergillus terreus* in itaconic acid production from glucose. Biotechnol Lett 14:201-206.

Park YS, Ohta N, Okabe M (1993) Effect of dissolved oxygen concentration and impeller tip speed on itaconic acid production by *Aspergillus terreus*. Biotechnol Lett 15:583-586.

Park YS, Itida M, Ohta N, Okabe M (1994) Itaconic acid production using an air-lift bioreactor in repeated batch culture of *Aspergillus terreus*. J. Ferment. Bioeng. 77:329-331.

Yahiro K, Takahama T, Park YS, Okabe M (1995) Breeding of *Aspergillus terreus* mutant TN-484 for itaconic acid production with high yield. J. Ferment. Bioeng. 79:506-508.

Yahiro K, Shibata S, Jia, SR, Park Y, Okabe M (1997) Efficient itaconic acid production from raw corn starch. J. Ferment. Bioeng. 84:375-377.

Riscaldati E, Moresi M, Petruccioli M, Federici F (2000) Effect of pH and stirring rate on itaconate production by *Aspergillus terreus*. J. Biotechnol. 83:219-230.

Dwiarti L, Otsuka M, Miura S, Yaguchi M, Okabe M (2007) Itaconic acid production using sago starch hydrolysate by *Aspergillus terreus* TN484-M1. Bioresource Technol 98:3329-3337.

Shin WS, Lee D, Kim S, Jeong YS, Chun GT (2013) Application of scale-up criterion of constant oxygen mass transfer coefficient (K_La) for production of itaconic acid in a 50 L pilot-scale fermentor by fungal cells of *Aspergillus terreus*. J. Microbiol. Biotechn. 23:1445-1453.

Gao Q, Liu J, Liu L (2014) Relationship between morphology and itaconic acid production by *Aspergillus terreus*. J Microbiol Biotechnol 24:168-76.

Shin WS, Park B, Lee D, Oh MK, Chun GT, Kim S (2017) Enhanced production of itaconic acid through development of transformed fungal strains of *Aspergillus terreus*. J. Microbiol. Biotechnol. 27:306-315.

Molnár ÁP, Németh Z, Kolláth IS, Fekete E, Flipphi M, Ág N, Soós Á, Kovács B, Sándor E, Kubicek CP, Karaffa L (2018) High oxygen tension increases itaconic acid accumulation, glucose consumption, and the expression and activity of alternative oxidase in *Aspergillus terreus*. Appl. Microbiol. Biotechnol. 102:8799–8808.

Supplementary Table S7: Reference literature containing input data used for **Figure 4** (trilateral relationship between carbon source concentration, Mn(II) concentration and yield of citric acid).

Clark DS, Ito K, Horitsu H (1966) Effect of manganese and other heavy metals on submerged citric acid fermentation of molasses. Biotechnol. Bioeng. 8:465-471.

Hossain M, Brooks JD, Maddox IS (1984) The effect of the sugar source on citric acid production by *Aspergillus niger*. Appl. Microbiol. Biotechnol. 19:393-397

Papagianni M, Mattey M, Kristiansen B (1994) Morphology and citric acid production of *Aspergillus niger PM 1*. Biotechnol Lett. 19:929-934.

Kontopidis G, Mattey M, Kristiansen B (1995) Citrate Transport during the citric acid fermentation by *Aspergillus niger*. Biotechnol Lett. 17:1101-1106.

Papagianni M, Mattey M, Kristiansen B (1998) Citric acid production and morphology of *Aspergillus niger* as functions of the intensity in a stirred tank and a tubular loop bioreactor. Biochem. Eng. J. 2:197-205.

Papagianni M, Mattey M, Kristiansen B (1999) The influence of glucose concentration on citric acid production and morphology of *Aspergillus niger* in batch and culture. Enzyme Microb. Technol. 25:710-717

Papagianni M, Mattey M, Berovic M, Kristiansen B (1999) *Aspergillus niger* morphology and citric acid production in submerged batch fermentation: effects of culture pH, phosphate and manganese levels. Food Technol. Biotechnol. 37:165-171.

Papagianni M, Mattey M (2006) Morphological development of *Aspergillus niger* in submerged citric acid fermentation as a function of the spore inoculum level. Application of neural network and cluster analysis for characterization of mycelial morphology. Microb. Cell Fact. 5:3

Papagianni M (2006) Quantification of the fractal nature of mycelial aggregation in *Aspergillus niger* submerged cultures. Microb. Cell Fact. 5:5.

Supplementary Table S8: Reference literature containing input data used for **Figure 5** (trilateral relationship between carbon source concentration, Mn(II) concentration and yield of citric acid).

Krull S, Hevekerl A, Kuenz A, Prüße U (2017) Process development of itaconic acid production by a natural wild type strain of *Aspergillus terreus* to reach industrially relevant final titers. Appl Microbiol Biot 101:4063-4072.

Saha BC, Kennedy GJ, Qureshi N, Bowman MJ (2017) Production of itaconic acid from pentose sugars by *Aspergillus terreus*. Biotechnol Progr 33:1059-1067.

Karaffa L, Díaz R, Papp B, Fekete E, Sándor E, Kubicek CP (2015) A deficiency of manganese ions in the presence of high sugar concentrations is the critical parameter for achieving high yields of itaconic acid by *Aspergillus terreus*. Appl Microbiol Biot 99:7937–7944.

Batti M, Schweiger LB (1963). Process for the production of itaconic acid. US Patent 3078217A

Nubel RC, Ratajak EJ (1962). Process for producing itaconic acid. US Patent No. 3,044,941
